# Recent Progress in the Oral Delivery of Therapeutic Peptides and Proteins: Overview of Pharmaceutical Strategies to Overcome Absorption Hurdles

**DOI:** 10.34172/apb.2024.009

**Published:** 2023-08-26

**Authors:** Sonal Mehrotra, Pavan Kalyan BG, Pawan Ganesh Nayak, Alex Joseph, Jyothsna Manikkath

**Affiliations:** ^1^Department of Pharmaceutics, Manipal College of Pharmaceutical Sciences, Manipal Academy of Higher Education, Manipal 576104, Karnataka State, India.; ^2^Department of Pharmacology,Manipal College of Pharmaceutical Sciences, Manipal Academy of Higher Education, Manipal 576104, Karnataka State, India.

**Keywords:** Biologics, Peptides, Proteins, Oral delivery, Absorption, Permeation enhancement, Nanoparticles

## Abstract

**Purpose:**

Proteins and peptides have secured a place as excellent therapeutic moieties on account of their high selectivity and efficacy. However due to oral absorption limitations, current formulations are mostly delivered parenterally. Oral delivery of peptides and proteins (PPs) can be considered the need of the hour due to the immense benefits of this route. This review aims to critically examine and summarize the innovations and mechanisms involved in oral delivery of peptide and protein drugs.

**Methods:**

Comprehensive literature search was undertaken, spanning the early development to the current state of the art, using online search tools (PubMed, Google Scholar, ScienceDirect and Scopus).

**Results:**

Research in oral delivery of proteins and peptides has a rich history and the development of biologics has encouraged additional research effort in recent decades. Enzyme hydrolysis and inadequate permeation into intestinal mucosa are the major causes that result in limited oral absorption of biologics. Pharmaceutical and technological strategies including use of absorption enhancers, enzyme inhibition, chemical modification (PEGylation, pro-drug approach, peptidomimetics, glycosylation), particulate delivery (polymeric nanoparticles, liposomes, micelles, microspheres), site-specific delivery in the gastrointestinal tract (GIT), membrane transporters, novel approaches (self-nanoemulsifying drug delivery systems, Eligen technology, Peptelligence, self-assembling bubble carrier approach, luminal unfolding microneedle injector, microneedles) and lymphatic targeting, are discussed. Limitations of these strategies and possible innovations for improving oral bioavailability of protein and peptide drugs are discussed.

**Conclusion:**

This review underlines the application of oral route for peptide and protein delivery, which can direct the formulation scientist for better exploitation of this route.

## Introduction

**Figure 1:**
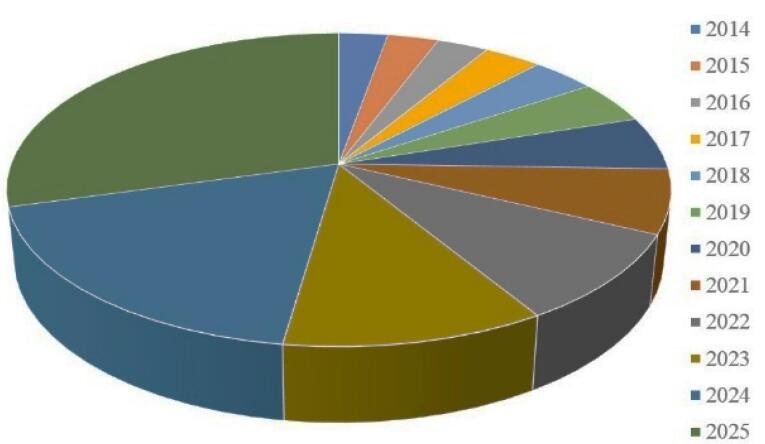
U.S. Market size analysis for peptides from 2014-2025

 Biologics include an array of complex molecules comprising carbohydrates, nucleic acids, proteins, peptides, cells, tissues and other products derived from living cells or biological processes.^1^ These have revolutionized the treatment of a wide range of diseases such as diabetes, hypertension, inflammatory disorders (rheumatoid arthritis, asthma, endometriosis and inflammatory bowel disease [IBD]) and cancer.^2^ They are potential therapeutic agents for combating these conditions due to their varied activities and interaction in many diseases. Peptides and proteins (PPs) constitute crucial classes of biological products. They are prospective therapeutic agents for combating a variety of pathologic conditions due to their high selectivity and efficacy and lesser adverse effects compared to small molecules. Peptides are polypeptide chains with 50 or fewer amino acids and a relative molecular mass not exceeding 5000 Da, with a high degree of secondary structure.^3^ In contrast, proteins are macromolecules that are made up of amino acids. They include fifty or more amino acids, with hundreds to thousands of these amino acids joined together as long chains of polymer to design a protein.^4^ The market for peptide and protein therapeutics is now projected to cross more than 40 billion USD per year, contributing to approximately 10% of the pharmaceutical franchise. This sector is developing at a considerably rapid pace, and is set to capture the major portion of the market in the near future.^5^ Approximately 75% of therapeutic macromolecules including PPs are delivered by parenteral methods, resulting in high costs and poor patient compliance. A survey on the market size of the therapeutic peptide distribution in the United States predicted a significant growth in peptide distribution by 2025 as shown in Figure 1.^6^

 For commercially available protein formulations, non-invasive delivery routes such as pulmonary, ophthalmic, nasal, rectal, buccal, vaginal and other routes have been investigated.^7-10^ However, oral administration is the most convenient and preferred mode of therapeutic delivery, with the highest patient compliance, therapeutic simplicity, and low cost of production.^11-14^ Due to significant barriers in the gastrointestinal tract (GIT) such as acid-catalyzed hydrolysis, proteolytic degradation by enzymes, inability to cross the membrane, first-pass metabolism during transfer across the absorption barrier, high molecular weight ( > 700 Da), and hydrophilicity, the oral delivery of PPs is difficult to achieve.^15-20^ Despite these significant constraints and roadblocks, substantial research has been done to develop advanced delivery methods that enable the oral delivery of therapeutic PPs. Many alternative techniques have been examined, and some of them have proven to be quite successful by making it to the market, while others are still in various phases of development.^21-25^ In this article, we highlight the various pharmaceutical approaches to improve oral bioavailability of PPs, including the use of absorption enhancers, enzyme inhibition, chemical modification, particulate delivery, site-specific delivery in the GIT, use of membrane transporter, lymphatic targeting and other novel approaches. We overview this from the standpoint of the physiology of the GIT, possible intestinal transport mechanisms of macromolecules and the major physiological barriers of macromolecular delivery.

## The gastrointestinal tract

**Figure 2:**
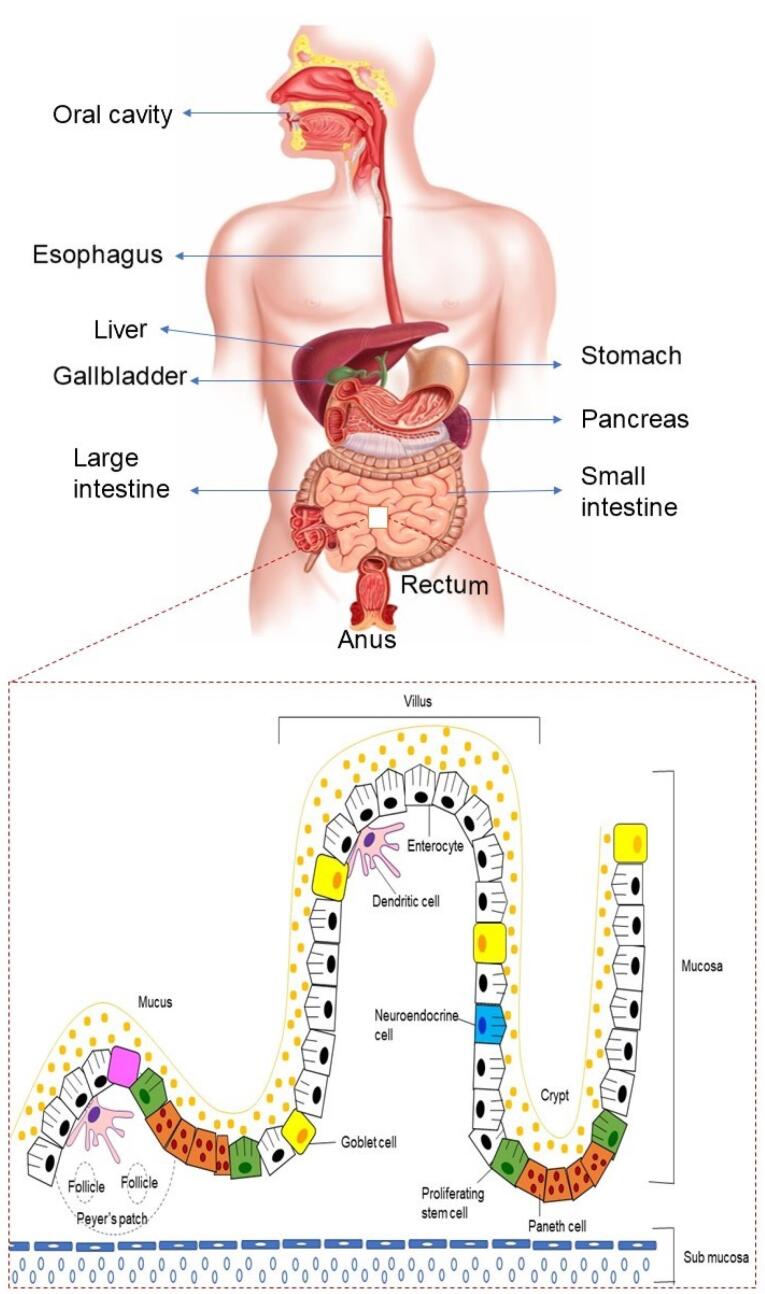
Physiology of GIT

 The human gastrointestinal system is made up of exclusive organs and is segmented into two parts. The mouth, oesophagus, stomach, duodenum, jejunum, and ileum are all part of the upper GIT, while the colon, rectum, and anus are all part of the lower GIT.^26^ Figure 2 depicts the physiology of the GIT. The pH of the upper part of the GIT varies, for instance, the stomach is acidic (pH 1.5-3.5), while it rises considerably in the duodenum (pH 5-6), distal jejunum, and ileum (pH 7-8), and then progressively declines upon reaching the colon (pH 6). These pH levels have been found to demonstrate inter-individual variations.^27,28^

 Peristalsis starts by allowing the ingested food from pharynx, through the esophagus and eventually into the stomach. The bolus is broken down in the stomach by gastric acid and digestive enzymes, transporting the digested material (chyme), to the duodenum via the pyloric sphincter. This complex process facilitates the disintegration of macromolecules like carbohydrate, lipid, protein, fiber into smaller digestible components in the small intestine to allow for nutritional absorption. The organs of digestion include salivary glands, pancreas, liver, and gallbladder. The luminal contents now reach the large intestine and are prepared for evacuation via the rectum and anal canal.^26^

 The digestive tract contains an architecture created from a variety of layers, together with the inner membrane layer of the GIT, that consists of absorptive cells and secretory epithelial cells, to coordinate the digestion processes. Other layers of GIT include the submucosal layer, smooth muscle layer, and serosal layer.^26^ Enterocytes, goblet cells, and M cells are among the cell types found in the intestinal epithelial cell layer.^29^ Goblet cells release the main component of mucus, and epithelial enterocytes have a role in ion, water, sugar, amino acid, or vitamin B12 absorption. M cells, found in Peyer’s patch follicle-associated epithelium, transport soluble macromolecules, particulates, and antigens from the lumen to immune system cells.^30^ The microvilli present in the mucosal cells of small intestine helps in promoting the drug absorption by providing a large surface area. Numerous enzymes present in the GIT, *viz*., trypsin, carboxypeptidase, pepsin, lipase, and amylase contribute towards the poor availability of biological macromolecules leading to their poor oral absorption. For instance, in the stomach, pepsin breaks peptide bonds resulting in a mixture of intermediate proteins and peptides, and amino acids. These are delivered into the duodenum where the action of pancreatic proenzymes break down these products into di- and tripeptides and amino acids.^31^

 The composition of GI fluids impacts the dissolving behavior and key factors for permeability in the GI tract by determining the therapeutic concentration. The solubility and dissolution rate of hydrophobic medicines in the stomach can be affected by the composition of bile salts and pancreatic enzymes. The general composition of intestinal fluids fluctuates throughout intestinal transit because of digestion and absorption activities.^32^

## Transportation mechanisms

 For peptides to get absorbed, first they should pass through the intestinal epithelial membrane. The epithelial barrier of the small intestine provides an even larger hurdle to oral protein therapeutic administration than peptide breakdown. A single layer of columnar epithelial cells supports the barrier, which is maintained by the lamina propria and muscularis mucosa.^33^ Drug absorption in the intestine can proceed by passive diffusion (either paracellular or transcellular) or by active diffusion with the help of transporters. The physicochemical and biological properties of the membrane in different parts of GIT decide the transport mechanism of the drug.

**Figure 3:**
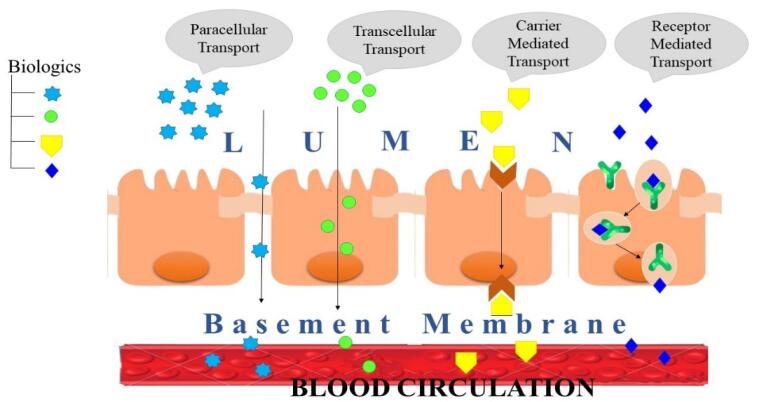
Macromolecule transportation mechanism

 Molecules traverse the membrane in four different ways: paracellular, transcellular, carrier-mediated, and receptor-mediated transport (Figure 3).

###  Paracellular transport

 The transfer of drugs across the junctions between GI epithelial cells is known as paracellular transport. In terms of drug absorption, this route plays a minor role. Drugs that are polar and hydrophilic in nature are expected to get transported by this route. Peptides are thought to pass through aqueous routes, including the paracellular and aqueous pore pathways.^34^ Rigid junctions act as the rate limiting factor for the transportation. Therefore, it can be said that these rigid junctions are one of the considerable factors while determining permeability by the paracellular transport.

 There are two essential requirements to overcome the rate limiting hurdle. Firstly, paracellular transport is unchangeable, meaning it is totally dependent on local concentration gradients (passive route). Secondly, mucosal permeability can be influenced by methods apart from tight junction control.^35^ There are three parts to the junctional complex: (1) the outer surface of macula adherens, popularly known as spot desmosome; (2) zonula adherens (belt desmosomes) in the center spot, forming a bridge between the outer and inner part; and (3) zonula occludens, the segment of the junctional complex closest to the lumen, also called ‘occluding’ or tight junctions (TJ).^36^ TJs are made up of a complicated sequence of aquaporin proteins that are divided into transmembrane proteins and cytosolic proteins. Plaque proteins, particularly zonula occludens-1 (ZO-1) and zonula occludens-2 (ZO-2), work along with regulatory proteins to create an integrity, and regulate the permeation across the tight junction.^37^

###  Transcellular transport

 The passage of drugs across the GI epithelium via passive diffusion, active diffusion or endocytosis is referred to as transcellular transportation. It is the predominant route for drug transport.

 The three stages involved in medication transcellular transport are: (1) drug absorption is hindered by the permeation of the GI epithelial cell membrane, which acts as a lipoidal barrier; (2) movement inside and between cells (cytosol); (3) lateral or basolateral membrane permeation.^38^ The permeability of a peptide by this route will depend upon numerous physicochemical features of macromolecules such as molecular structure, molecular mass, partition coefficient, surface charge, hydrogen bond interaction, and orientation.^39^ Endocytosis mechanisms, such as phagocytosis, pinocytosis, and receptor-mediated endocytosis, are commonly used to transport macromolecules in the small intestine.^40^ The transportation of macromolecules through transcellular route does not depend on transmembrane channels.^35^ Interestingly, investigations reveal that transcellular absorption declines considerably throughout the colon (small intestine > ascending colon > transverse colon), but the paracellular pathway has no such gradient.^41^ This pathway is only suitable for the transfer of lipophilic substances with a low molecular weight.^39^

 The major intestinal cells for transport are enterocytes and M cells. The former represent the major portion of GIT whereas the latter are predominantly found in a smaller percentage of intestinal epithelium (mainly within the epithelium of Peyer’s patches).^42^ The high ability of endocytosis of M cells makes them a potential portal to deliver PPs orally.^43,44^ M cells use a variety of mechanisms to adopt macromolecules and particles including fluid phase endocytosis, absorptive endocytosis, and phagocytosis.^45^ Various ligands, such as salmonella extract, Ulex europaeus agglutinin 1 ligand, invasin, and immunoglobulin A, can be attached to the surfaces of the particles to improve their absorption by the intestinal M cells.^46^ On the other side, energy dependent mechanisms like macropinocytosis, clathrin- or caveolae-mediated endocytosis and clathrin and caveolae-independent endocytosis, help in the transportation of nanoparticles (NPs).^47^

###  Carrier mediated transport

 Drug molecules are transported with the help of carrier systems across the cell membrane and then delivered into systemic circulation.^34^ The process requires adenosine triphosphate in the form of energy to promote the uptake of macromolecules with the help of the carrier. Binding of the carrier to the target molecule depends upon various factors. Drug-carrier complex crosses the intestinal membrane, even against the concentration gradient. For instance, small di/tripeptides (such as lactam antibiotics and angiotensin converting enzyme inhibitors), monosaccharides, and amino acids are transported by this mechanism.^48^

###  Receptor mediated transport

 In receptor-mediated transport, protein and peptides act as receptor for the ligand present on the cell surface or itself act as the receptors on the cell surface. Clathrin-mediated endocytosis and caveolae-mediated endocytosis are some effective pathways for biological macromolecule uptake.^49^ Clathrin-mediated endocytosis results in the formation of vesicles covered with clathrin protein (120 nm), which then fuse with early endosomes.^49^ This technique works well with particles under 200 nm. With increasing particle size, caveolae-mediated internalization is the primary route of entrance, especially for particles larger than 500 nm. Caveolae-mediated internalization is a non-specific absorption mechanism that results in the production of caveolae-coated vesicles (50-80 nm) that later undergo direct exocytosis due to their ability to avoid early endosomes.^49^ The most notable distinction between clathrin-mediated and caveolae-mediated endocytosis is that the former involves the invagination of clathrin-coated pits, whereas the latter involves the formation of a large number of buds on the membrane.^50^

## Gridlocks to oral delivery of therapeutic peptides and proteins

 Despite the fact that oral delivery of PPs has piqued the interest of drug manufacturers and funding bodies, there are a number of factors hindering the oral absorption of PPs, including GIT instability, enzymatic degradation, acid hydrolysis, impermeability of macromolecules across intestinal epithelial membrane, and difficulty in formulation. The cell lining, comprising outer membrane of cell and tight intersections between adjoining cells is largely responsible for the physiological barrier, albeit the mucus membrane and many of efflux transporters may likewise assume a part in controlling medication absorption.^51^ These barriers, however, are also the first line of protection against toxins, antigen, and pathogens. To overcome hurdles to oral distribution of PPs, it is important to thoroughly comprehend the physiological and physicochemical aspects of the formulation. The absorption cycle is described below to recognize the hindrances that therapeutic macromolecules should defeat during their uptake into the blood stream following oral ingestion.

###  Physiological barriers 

 Following oral administration, the drug may encounter a number of physiological barriers that affect PPs absorption, including pH gradient, gastric emptying rate, intestinal transit duration, surface area, permeation across the epithelial membrane, and the expression of intestinal enzymes and transporters.^32^

####  pH gradient

 Every part of the GIT has a particular pH, which is controlled by an assortment of elements like the presence of food, various ailments, prolonged stress, life span, and sex. In healthy humans the pH is around 1.5-3.5 (acidic) in stomach, which increases to pH 5-6 (basic) in the duodenum because of carbonate and bile juice neutralization, and rises to pH 7-8 in the distal jejunum and ileum.^27,52^ The impact of food is the most powerful physiological stimulant to the production of acid by stomach and most likely, pepsinogens.^53^ Lower and higher pH levels are associated with greater caloric meals before and after meals, respectively. The extremes of pH changes were reduced by frequent feeding.^54^ High protein diets, on the other hand, result in greater hydrogen ion concentrations before meals and lower values after meals.^55^ Furthermore, individual differences in colonic pH may be due to personal dietary preferences. Age has limited influence on GI pH, indicating that the GI pH state can remain relatively constant throughout life.^56^ In any case, pH of the stomach is significant following child birth and soon settles towards the normal pH of 1 to 3.^57^ Healthy old adults aged above 70 have considerably lower stomach pH but much higher duodenal pH than healthy younger people aged around 50.^58^ Diseases including IBD, ulcerative colitis, and gastrointestinal malignancies can dramatically alter the pH of the GIT. In the colon of patients with active ulcerative colitis, the intraluminal pH level was observed to be low.^59^ While IBD does not affect the pH of the stomach or small intestine, it tends to lower the pH of the colon.^60^ The pH of the colon in patients of Crohn’s disease is lower than in healthy people.^61^

 The complex pH conditions in the GIT may cause structural changes or protein breakdown leading to therapeutic deprivation. Proteins are frequently persistent at pH levels near their isoelectric point (pI). As a result of pH-induced unfolding, certain proteins may be rendered inactive in stomach juices.^62^ For instance, pepsin has the greatest potential to degrade at pH 2-3 but is entirely inert at pH 5.^32^

####  Enzymes

 Proteolytic compounds (fundamentally trypsinogen and chymotrypsinogen, and their dynamic forms, trypsin and chymotrypsin), amylolytic proteins (pancreatic amylase), and lipolytic enzyme (lipase) are the three kinds of pancreatic catalysts.^63^ These enzymes break down proteins into peptides, which eventually get broken down into amino acids.^64^ Protein entry might cause the cells lining of stomach to secrete pepsins, by gastric mucosa. By hydrolyzing the peptide bond, pepsin may break down proteins into smaller peptide fragments.^65^ Peptidases found in the microvilli of intestinal epithelial cells, such as aminopeptidase and dipeptidyl peptidases 3 and 4, digest peptides of up to 10 amino acids, whereas intracellular peptidases digest dipeptides.^66,67^ The pancreas secretes a variety of degradable biocatalyst in the small intestine, including trypsin, chymotrypsin, carboxypeptidase, and elastase.^68^ The amino acids generated by enzymatic breakdown and other nutrients released by food digestion are absorbed into the systematic circulation from the small intestine, which is aided by the mucosal folds, villi, and the large surface area of the microvilli. The large intestine is home to 700 different bacterial species that help with digestion and absorption of the leftover food from the small intestine. Also, absorption of macromolecules in large intestine is negligible.^69^ As a result, protecting the stability of PPs in the GIT is one of the most essential conditions for the effective absorption of oral PPs.

####  Mucus

 Even if the drug can get beyond the pH barrier and the enzyme barrier, the mucosal barrier in the small intestine keeps the drug away from encountering epithelial cells. The surface area of human GI mucosa is at least 200 times that of the skin.^70^ This mucus barrier activity restricts drug penetration in two ways, firstly, by restricting positively charged peptides attachment to adversely charged mucin fibres, and secondly, by creating a barrier to the lattice shape of membrane during absorption.^71,72^

 In different parts of the GIT, the mucus layer thickness varies substantially. Mucus is made up of several different components. Complex carbohydrates, polypeptides, salts, antibodies, microbes, and cell detritus round out the list of active components, with mucin glycoprotein being the most significant.^73^ The mucus gel layer is made up exclusively of glycoproteins (mucins), and may operate as an obstacle to drug absorption by keeping the aqueous layer undisturbed or through associations among the diffusing compounds and mucus layer constituents.^74,75^ Multiple obstacles to medication transport into the submucosal tissue are created by mucus.^76^ The high viscosity reduces PP diffusivity via mucus, which has a direct impact on PP residence duration in the small intestine. The typical mucus turnover period in the gut is about 50-270 minutes, leading to clearance of trapped particles in the mucus layer and therefore reducing particle adhesion and holding duration.^77^

####  Epithelial barrier

 The main cells including epithelial and M cells helps in the transportation across the transmembrane.^78^ Therapeutic proteins taken orally must pass through phospholipid film prior to entering the fundamental course, once crossing the gastro-intestinal mucosa.^79^ Enterocytes, mucus production, Paneth cells, and M cells for absorption, production, enzyme secretion, and particle transport, respectively are among the different kinds of cells found in the intestinal epithelia.^80^ The existence of a transporter on the epithelial surface regulates the permeability of macromolecule in the small intestine via a paracellular or transcellular route.^32^ In terms of genetic differentiation pathways and unique functions, the intestinal epithelium is divided into two kinds of cells: absorptive and secretory cells. Enterocytes, which make up 90% of the small intestinal epithelium, serve as absorptive cells in the small intestine. Cup cells and M cells, after all, have long been recognized as epithelial cells that are neither absorptive nor secretory. Due to existence of rigid junctions between two neighboring layers of epithelial cells, the impervious intestinal layer serves as a guardian to biologics as well as an adsorbing and productive surface.

####  Efflux pumps

 Efflux pumps belong to domain of ATP binding cassette and show their presence on the anterior end of cells and are mainly responsible for multidrug resistance.^81^ P-glycoprotein I (PGP-I) is one example of an efflux pump. PGP-I can pump drugs and peptides back into the GI lumen after they have been absorbed. PGP-I is known to be a substrate for linear lipophilic and cyclic peptides macromolecules.^82^

###  Physicochemical factors

####  Molecular weight and size of molecules

 The molecular weight and size of the macromolecule have a big role in drug diffusion over the epithelial layer. Small molecules might readily move over the concentration gradient passively, while the entry of extremely large biomolecules is restricted. The main reason behind this obstacle is the lack of energy that is caused by the difference in the concentration gradient leading to limited entry/insertion of molecules across the membrane.^83^ With increasing molecule size, drug diffusion reduces dramatically. Molecular weight (MW) has been shown to affect the mucosal absorption of a variety of hydrophilic substances in many studies.^84,85^ However, cyclic peptides with a large molecular weight have a greater rule of violation, and some peptides violate the Lipinski rule of five (RO5) criteria by being available orally. Early studies suggest that this chameleon characteristic of many macromolecules (high molecular weight) is closely related to RO5 blueprints as found in orally accessible peptide medicines. A chameleon molecule may change its shape and polarity with respect to the outer environment i.e., hydrophilic or lipophilic, due to its intermolecular hydrogen bonding arrangement.^86^ The permeability coefficient is inversely proportional to molecular weight. As seen in an example, the permeability coefficient of fluorescein isothiocyanate dextran decreases as MW increases.^87^ With MW greater than 300 Da, absorption often reduces exponentially. The MW of therapeutically utilized biologics varies greatly, starting from hundreds to hundreds of thousands ( < 500-100 000 Da) making juxtaposition difficult.^88,89^ Human *in vivo *permeation experiments revealed that peptides like protirelin (MW: 362 Da) and oxytocin (MW: 1007 Da) could pass the buccal mucosa barrier, while buserelin (MW: 1239 Da) and calcitonin (MW: 3500 Da) could not.^90^ Merkel et al demonstrated that using an absorption enhancer increases the bioavailability of high molecular weight peptides. Simultaneous feeding of compounds has also been found to improve the performance of other epithelia. Buccal peptide delivery allows clinically appropriate dosages to permeate, for example, insulin, oxytocin, vasopressin analogues, protirelin, and octreotide.^91^

####  Molecular charge

 The influence of charge on passive diffusion of drugs is well understood. Passive diffusion of charged molecules is less effective than passive diffusion of uncharged ones.^88^ At the pI of biomolecules, they appear as zwitterions and thus have a detrimental influence on membrane permeability.^92,93^ In the prediction of oral absorption, the charge over the surface of peptide molecules is a key factor. Additionally, altering the pH of the medium, results in change in the degree of ionization, charge density, and permeability of the peptide.^89^ The positively charge peptide molecules get attracted towards the negatively charge epithelium membrane at biological pH or above the pI and vice versa.^90^ The membrane is non discriminating to either ion at the isoelectric point.^91^ In physiological conditions, however, changing the pH of biological systems can reduce the stability, and increases the catalytic degradation/ breakdown.^92^

####  Lipophilicity

 As drug molecules must permeate the lipid bilayer of cellular membranes, including those of enterocytes, lipophilicity of a drug molecule is a key problem in the design of dosage forms. As a result, drug molecules should be lipophilic in order to facilitate absorption. Based on water solubility and intestinal membrane permeability, the FDA proposed the Biopharmaceutics Classification System (BCS) and associated guidelines which divide bioactive constituents into four categories.^93^ As a result, peptide drugs are frequently categorized in BCS class III and BCS class IV having low-permeability, high-solubility and low permeability, low solubility properties, respectively. Thus, macromolecules face severe constraint during permeation. Some changes have been used to improve lipophilicity and absorption through the passive diffusion pathway, such as blocking the C-terminal by cyclization, amide production, or esterification of proteins.^94^ Although the octanol–water partition coefficient is a simple metric that can predict mucosal permeability, it does not necessarily correlate with peptide absorption since peptide bioavailability changes parabolically with lipophilicity.^95^

####  H bond donor and H bond acceptor

 The use of H-bond donors or acceptors to strengthen protein-ligand interactions frequently results in no net increase in binding affinity.^96^ By studying physicochemical computational modelling, it was shown that MW and hydrogen bond acceptors have grown significantly, whereas lipophilicity and hydrogen bond donors have experienced relatively minor increases. A large variety of hydrogen bond donors have been dubbed the “enemy of medicinal chemists” because of their potential to induce poor permeability, absorption, and bioavailability.^97^

####  Aggregation

 Peptide aggregation is a frequent and troublesome phenomenon that occurs at nearly every stage of biological drug development.^98^ Aggregation can take various forms and refers to a variety of mechanisms in which peptide molecules join to create bigger species with numerous polypeptide chains. They can develop because of non-covalent polypeptide chain association or covalent chain linkage. Aggregation is reversible in certain circumstances but practically irreversible in others. In either instance, it decreases the physical stability of the peptide, resulting in a loss of activity as well as other serious issues including toxicity and immunogenicity.^99^

## Strategies for improving oral delivery of proteins and peptides

###  Absorption enhancers

 Absorption enhancers are chemicals that are given in conjunction with a therapeutic protein or peptide to help it absorb effectively. They reversibly break or eliminate the epithelial roadblock with minimal harm to normal tissues, making a peptide permeate across the intestinal membrane and reaching the systemic circulation.^100^ In certain cases, increased intestinal permeability has been linked to acute epithelial injury. The research, on the other hand, shows that some absorption enhancers can improve peptide penetration in a reversible manner without any tissue injury or showing hazardous consequences.^101^ There are different approaches by which absorption enhancers create a temporary breach in the epithelial cell barrier in the gut, allowing proteins or peptides to be absorbed. These processes include structural changes in epithelial cell membranes that lead to increased passive diffusivity of macromolecule either by penetrating across the cells (i.e., paracellular pathway) or by transporting between epithelial cells.^102^ Absorption enhancers are classified according to their chemical structure and method of action. Medium-chain fatty acids (caprylate, caprate, and laurate, respectively), can improve the permeability of hydrophilic compounds through paracellular route, with caprate > laurate > caprylate is the sequence of increasing absorption *in vivo.*^103,104^

 Another sort of absorption enhancer is lectins. They are proteins that detect sugar complexes linked to proteins and lipids and bind to them. They act by binding to the luminal surface of small intestine which lead to increase the permeation of peptides by vesicular transport and reaching the systemic circulation.^105^

 Surfactants (detergents) disrupt proteins and lipids at membranes, allowing chemicals like therapeutic PPs to move more freely between cells. Anionic and non-ionic detergents are examples of this. Anionic surfactants are more effective in increasing transepithelial permeability than non-ionic surfactants.^106^ Tetradecyl maltoside (TDM), a soluble surfactant, improves the bioavailability of the anticoagulant medication enoxaparin (given orally) by transiently lowering transepithelial electrical resistance in C2BBel cell extracts.^107^

 Permeation enhancers come in a variety of forms (chelating agents, surfactants or detergents, bile salts, salicylates, toxins, venom extract, fatty acids, various polymers), each with its unique mode of action and some examples are shown in Table 1.

**Table 1:** Common absorption/penetration enhancers and their mechanisms of action

**Class**	**Example**	**Mechanism of action**	**Study involved**	**Reference**
Chelating agent	EGTA (Egtazic acid)	TJ opening & increase penetration via paracellular route (Calcium and magnesium complexity)	Caco-2 cell culture model	^108^
Fatty acid	Medium chain glycerides (CapMul MCM)	Increase in marker molecular permeability	Chamber technique (*In vitro*)	^109^
Toxins	Zonula occludens toxin (ZOT)	Actin polymerization (opening of tight junction) is induced by interaction with the zonulin surface receptor	Caco-2 cell monolayer	^110^
Bile Acids	Sodium deoxycholate	Endogenous surfactant; act by terminating the lipid portion beyond CMC	Rat	^111^
Surfactant	Anionic (sodium dodecyl sulfate and sodium dioctyl sulfosuccinate)	Cause membrane disturbance by depleting membrane proteins or lipids, as well as phospholipid acyl chain disruption.	*In vitro* dioctyl sulfosuccinate perturbation (Caco-2 cells)	^112^
Polymers	Anionic polymer (carbomer)	Synergistically cause enzyme inhibition and calcium reduction outside the cell leading to opening of tight junction. Polymers can also help to decrease transepithelial electrical resistance.	*In vitro* (Caco-2)	^113^
	Cationic polymer (chitosan)	Interacts reversibly with elements of rigid junctions, causing the paracellular pathways to expand.	*In vitro* (Caco-2)	^114^

 Due to permanent epithelium damage, several types of absorption enhancers have fallen off the radar in recent years.^115^ Surfactants like sodium dodecyl sulphate (SDS) have been found to enhance the permeability of the GIT to hydrophilic substances while also altering cell shape and causing cell membrane damage.^116^ With even brief exposure to SDS, microvilli were shortened, and actin disbandment, structural separation of TJs, and damage to the apical cell membrane occurred.^106^ Several *in vivo* rat investigations back up the increased absorption and show that the harm is reversible.^112^ Bile salts like sodium cholate and deoxycholate have been regarded to be helpful in enhancing medication absorption; nevertheless, long-term use of these particles have demonstrated mixed results.^117^ Studies on the safety and toxicological studies of common intestinal absorption enhancers have been given (Table 2).

**Table 2:** Safety and toxicological studies of intestinal absorption enhancers

**Absorption enhancer**		**Model**	**Dosing**	**Observations**	**Reference**
Medium chain fatty acid technology: Gastro-Intestinal Permeating Enhancement Technology (GIPET^TM^; Merrion Pharmaceuticals, Dublin): Solid-dose/ microemulsion-based	GIPET I (C10 and C12, ratio 1:2)	Canine	0.1, 0.3, 0.9 g/kg/day, up to 14 days	Emesis in some animals 1 h after administration of highest dose; No micro- or macroscopic changes in intestinal tissue with any dose	^118^
	GIPET II Mono/ diglycerides of C_8_ and C_10_	Canine	0.4, 2.0, 4.0 g for 7 days	No clinical pathology, histopathology or changes in body weight	
Medium chain fatty acid technology using sodium caprylate C_8_: Transient Permeability Enhancer (TPE^®^), Chiasma Ltd., Israel)		Cynomolgus monkey	Daily administration of capsule by intragastric intubation, for 9 months	No signs of toxicity; No changes in ECG, bodyweight, clinical, ophthalmological or hematological pathology	^119^
Surfactants: URB1480-URB1482 (sucrose-based); URB1419-URB1421 (lactose-based)		Calu-3 cells (airway epithelium)	0.03 to 4.5 mM	No remarkable cytotoxicity; reduction in cell viability to 70% with 4.5 mM of URB1481 alone	^120^
Bile salt: sodium deoxycholate (also a surfactant)		Mice	200 mg/kg, for 30 days	No significant different in tumor necrosis factor (TNF)-α levels (indicator of inflammation) compared to control; No weight loss; Temporary change in fecal quality; No permanent impairment of intestinal barrier function	^121^
1-Phenylpiperazine		Mice	60 mg/kg for 30 days	Weight gain significantly lesser than control group; No change in fecal quality; No permanent impairment of intestinal barrier function	^121^
Acyl carnitine: palmitoyl carnitine chloride		Caco-2 cells	0.01 to 1 mM	No remarkable decrease in cell viability	^108^
EGTA		Caco-2 cells	0.01 to 10 mM	No remarkable decrease in cell viability; concentrations ≥ 1 mM increased cell viability	^108^

 Permeation enhancers were used in another investigation to examine localisation and controlled release of permeation enhancers to minimise the unpredictability associated with peptide absorption. Tyagi et al^122^ utilised layering techniques to produce a multi-unit particulate system (MUPS) in which the active peptide (MEDI7219), permeation enhancers, and polymers are coated for peptide release. According to the findings, layering of peptide and permeation enhancers over sugar spheres was shown to increase interaction and simultaneous solubilization and exposure to the GIT. The distal small intestine and the proximal large intestine were determined to be the sites of absorption of MEDI7219 during the early studies.

###  Enzyme inhibition

 Orally administered proteins can have low bioavailability due to enzymatic breakdown in the GIT. Co-administration with protease inhibitors such as pancreatic inhibitor, soybean trypsin inhibitor, camostat mesylate, and aprotinin, which improve the bioavailability of orally given PPs by decreasing their enzymatic breakdown by trypsin or α-trypsin, is an alternative to structural modification.^123^ These enzyme inhibitors operate by interacting to the target biocatalyst in the intestine in a reversible/irreversible manner.

 Following represents a categorization of suppressive drugs depends on their structural makeup^124^:

1. Inhibitors which are independent of amino acids (e.g., phenylmethylsulfonyl fluoride)
2. Amino acids and altered amino acids (e.g., chymostatin, amastatin)
3. Peptides and modified peptides (e.g., N-acetyl cysteine)
4. Protease inhibitors (e.g., Aprotinin)

Other protease inhibitors, like chymostatin, Bowman–Birk blocker, aprotinin, improve insulin microsphere bioavailability by inhibiting digestion by digestive enzymes such as pepsin, trypsin, and –chymotrypsin.^125^ Polypeptide protease inhibitors like aprotinin have a large molecular weight, which enables for efficient formulation in long-acting oral dose forms such insulin-loaded polyvinyl alcohol-gel spheres.

 In numerous tests, puromycin, an aminopeptidase inhibitor, was found to improve the uptake of metkephamid (MKA), a robust derivative of met-enkephalin, through the rat gut. Because aminopeptidase participates in MKA metabolism during absorption, endopeptidase inhibitor (thiorphan) proved unsuccessful in preventing MKA metabolism. At high pH, amastatin was shown to inhibit the breakdown of the pentapeptide leucine (Leu)-enkephalin (YGGFL).^126^

 Altering the pH at the active site of the enzyme is an alternate technique of inhibiting enzymes.^127^ Most stomach enzymes including pepsin are active at a low pH ( < 2).^128^ As a result, if the gut pH rises, the biocatalyst loses its ability to break down the protein counterpart. Biocatalysts present in the small intestine work best at high pH, and thus lowering it can limit their activity.^129,130^

 These enzyme inhibitors do have some lane blockers. For a start, they can interfere with dietary peptide absorption and cause toxic shock if used for an extended period of time.^130^ Although systemic toxicity as well as damage to the mucosa surface are ruled out, pancreatic protease enzyme inhibitors still have hazardous potential due to the suppression of these digesting enzymes. Aside from disruption of nutritive protein digestion, a feedback regulatory inhibitor-induced increase of protease production is to be predicted.^131^ In rats and mice, suppressors like Bowman–Birk blocker, soybean trypsin blocker, and camostat have been used to explore this feedback regulation. They show that this feedback control causes pancreatic hypertrophy and hyperplasia in a short period of time. Furthermore, long-term use of the Bowman Birk blocker and soybean trypsin blocker results in the development of many proliferative hotspots, which frequently progress to aggressive malignancy.^132,133^

###  Chemical modification

 Changing the chemical structures of macromolecules is one way to improve their pharmacokinetic profile and make them more therapeutically useful pharmaceutical agents.^134^ The goal of performing chemical changes to biologics architecture seems to reduce undesired characteristics such as excessive sensitivity to enzyme degradation, inappropriate miscibility, or inadequate penetrability. Chemical modifications can potentially be utilised by reducing immunogenicity of administered therapeutic biologics. Protein modification can also take the form of direct alteration of the proteins’ exposed side-chain amino acid groups^135^ or modification of glycoproteins and glycoenzymes.^136^ The latter method has the advantages of being suitable even when the protein sample is not particularly pure and of not interfering with the natural structure of the protein. Prodrug synthesis, backbone alteration of protein, linking of peptide molecule to biodegradable and non-biodegradable polymers, structural alteration to be recognized by transporters, as well as cyclization are all examples of chemical modifications.

####  PEGlyation

**Figure 4:**
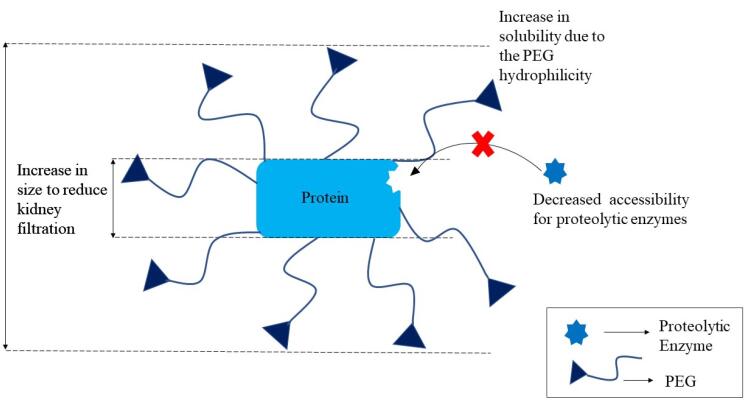
PEGylation for improving properties of proteins

 The technique of covalently attaching polyethylene glycol (PEG) molecules to the framework of a biologic to improve its pharmacokinetic characteristics and therefore turn it into a more effective therapeutic agent is known as PEGylation. Biologics are delivered orally using polymers such as PHPMA (poly (N-2-hyfroxypropyl methacrylamide)), POEGMA (polyoligo (ethylene glycol) methyl ether methacrylate), PNIPAm (poly(N-isopropylacrylamide)), PLGA (poly (lactic-co-glycolic acid)), PDEAM (poly(N,N-diethylacrylamide)), PLGA (poly(lactic-co-glycolic acid)) and PDEAM (poly(N,N-diethylacrylamide)). Biocompatibility, reduced toxicity, enhanced biological and circulatory half-life, and cheaper cost are some advantages of PEGylation.^137^ PEG molecules (associated to biomolecules) have an excellent capability to act as a barrier, preventing proteolytic enzymes from reaching and hydrolyzing the protein or peptide,as illustrated in Figure 4. PEGylation of targeted medication delivery has been proven in studies to reduce clearance and improve distribution of PPs.^138^

 The *in vitro* anticancer effectiveness was investigated by PEGylating siRNA lipoplexes by shutting B-lymphocyte triggered maturation protein (BLIMP-1) lymphoma cells. Post-insertion of PEG on premade lipoplexes was shown to have a lot of potential for concealing siRNAs and reducing the interaction with serum protein in this investigation. PEGylation resulted in stable 300 nm siRNA lipoplexes with a complexation efficiency of 80%.^139^

 The controlled release pattern of bovine serum albumin used as a model protein from a hydrogel-based matrix was investigated using chemically bridged chitosan-PEG derivatives. In simulated gastric fluid, the hydrogel was treated with or without enzyme. Because of reductive amination, PEG dialdehyde may inflate at basic pH and remain deflate in lower pH. The total release kinetics were found comparably sluggish in the first 2 hours but enhanced considerably at pH 7.4 with lysozyme over 12 hours. The findings demonstrated the behaviour of cross-linked CSPEG-H-CS hydrogel derivatives, indicating that they are appropriate for oral protein administration.^139^

 Surface modification of nanoliposomes using PEG and peptide medicines was investigated by Yazdi and coworkers.^140^ In their study, folic acid was conjugated to (methoxy polyethylene glycol-di-stearyl phosphatidyl ethanolamine) DSPE-PEG3400NH2, and liposomes were stabilised with mPEG-DSPE to improve stability under severe GIT conditions and serve as an efficient carrier for folate targeting. According to the findings, PEGylated phospholipids might increase the efficacy of insulin medication delivery when taken orally.

 When looking at the negative aspects of the PEGylation method, it becomes clear that it most obviously decreases the therapeutic effect of PEGylated macromolecules. The main cause of this is due to configurational alterations caused by PEG polymer coupling, which might result in intermolecular interactions among macromolecules and its target ligand disruption. There are increasing numbers of reports of delayed and rapid hypersensitivity reactions to PEG-containing compounds, as well as immunological reactions to PEG. Increased vacuolation has been seen in tissues of animals given PEGylated proteins. Another unanticipated result of PEGylation was that the addition of PEG resulted in increased viscosity. Kerwin’s group^141^ found that a combination of PEG and tumor necrosis factor (TNF) receptor1 had a viscosity five times greater than PEG or TNF receptor1 alone. PEG can be branched or single-chained.^142^

####  Pro-drug approach

 Prodrug is an active pharmacological moiety that has been chemically modified into an inactive form and upon administration is transformed into the dynamic form to express pharmacological activity. Intake of a prodrug requires activation, either chemically or by enzyme-mediated conversion to the parent drug. This stage separates the creation of prodrugs from that of ordinary drugs. This extra step is believed to have contributed to the pharmaceutical industry’s historical reluctance to use a prodrug strategy early in the development process; nevertheless, it is now widely acknowledged that this extra step also has a lot of promise.

 The use of a prodrug method might aid in the absorption of biomolecules including RNA, DNA, oligonucleotides, and proteins. This technique might be used to improve drug pharmacokinetics, achieve prolonged drug release, and reduce toxicity.^143^ The molecular revolution in biology and medicine has enabled a contemporary approach to prodrug design that incorporates molecular/cellular characteristics and is targeted at target molecules in the body. If the parent drug has constraints like poor miscibility, stability issues, inadequate permeation, and low half-life, this is an overriding strategy.^144^ Tanaka et al used the chemically modified TRH derivative lauryl-thyrotropin releasing factor (TRF) as an example. Conjugation of TRH with lauric acid dramatically enhanced TRH penetration into the upper small intestine. TRF was gradually converted to natural TRH in the brush-border membrane fraction, to describe the prodrug technique.^145^

 Bundgaard^146^ discussed various approaches to derivatization of peptides to produce prodrugs, including N-alkylation of peptide bonds to yield N-a-hydroxyalkyl derivatives, esterification to yield N-a-acyloxyalkyl derivatives, and making a-hyroxylglycine derivatives, as well as TRH delivery as a N-alkoxycarbonyl prodrug derivative are instances of the prodrug strategy for resolving delivery issues. Phenyl propionic acid was used to chemically modify (Lue5)-enkephalin into a prodrug, which was not only shown to enhance their permeability across Caco-2 but also stability.

 Peptide–drug conjugates (PDCs) are a specific category of prodrug in which a particular peptide sequence is covalently attached to a drug via a cleavable linker. Because the amino acid sequence may be selected to regulate both the physicochemical characteristics of the conjugate and the active targeting of a specific receptor on the tumour cell surface, peptides enable for a high degree of functionality to be included into PDCs. PDCs are biodegradable and do not provoke unwanted immunogenic responses since they are composed of amino acids and have short peptide lengths.^147^

 Self-assembling PDCs, in which individual conjugates have the potential to assemble nanostructures, are an emerging subgroup of PDCs that aims to combine the benefits of peptide-based prodrugs with those of a vehicular delivery strategy. PDCs effectively constitute their own drug delivery vehicle in this unique design, which can break down over time or in response to a specific stimulus and releasing the active medication.^148^

 Although prodrug techniques have been successful for modest chemically synthesized medications and few short-chain peptides, the structural complexity of peptides and the absence of innovative approach may restrict their use to peptides in general. The majority of peptide prodrug methods have been limited to modify a single functional group.

####  Peptidomimetics

 Peptidomimetics are meant to overcome some of the drawbacks of natural peptides, such as stability against proteolysis (activity duration) and low bioavailability. Peptidomimetics are substances whose essential components (pharmacophore) in 3D space imitate a natural peptide or protein while maintaining the capacity to interact with the biological target and generate the same biological effect.^149^ Their non-peptide moiety can be altered by adding cyclic peptides or non-natural amino acids or changing the backbone structure to improve bioavailability and half-life. As a result, properties that are often absent in natural peptides, such as greater receptor selectivity and decreased metabolic liabilities are improved, resulting in higher potency^150^ Peptide modifications can occur whether in the amino acid sequence or the amino acid branched groups, or both. Common procedures include N-alkylation which has been proven to improve peptide bioavailability and isosteric exchange of the amide link also helps in enhancing the activity.^151^

 N-alkylation boosts the total lipophilicity of the peptide while also inducing steric hindrance. Furthermore, during N-alkylation, the added alkyl group substitutes the hydrogen which was originally attached to the nitrogen atom of amide bond. The conformation of the peptide may be affected by the reduced hydrogen bonding capabilities. Cyclosporine is an example of this, it may be ingested orally and shows 29% bioavailability. In this, methyl group has alkylated the nitrogen atoms (amide bonds). Isosteric substitution of the amide bond is another peptidomimetics design method that has received a lot of attention.

####  Glycosylation

 Glycosylation is a widely protected mechanism in eukaryotic and bacterial posttranslational protein modifications.^152,153^ Glycosylation may be induced in the laboratory by chemically conjugating a carbohydrate molecule to another macromolecules like protein, fat, or nanoparticulate carrier. In this approach, a wide range of glycan structures may be produced. Carbohydrate moieties are added to protein to modify the structure and function by steric influences involving the intermolecular and intramolecular interactions, results in enhancing physicochemical properties, organelle localization, and target binding.^149,153,154^

 N-linked and O-linked glycosylation are two prevalent post-translational modifications. Sugar protein linkages to serine and threonine (“O-linked”) or arginine and asparagine residues (“N-linked”) are the most important in glycoprotein. Hyperglycosylation refers to the condition characterized by excessive glycosylation. Many of the same advantages of PEGylation apply to hyperglycosylation, including extended half-life, better solubility, and decreased immunogenicity. By shielding non polar domains on the protein surface implicated in weaker bonding coupling that induce accumulation, activity loss, and/or enhanced sensitivity, hyperglycosylated peptides enhanced its stability.^155^ However, steric hindrance may limit the action of hyperglycosylated therapeutic proteins.

###  Particulate delivery

####  Nanoparticles

**Figure 5:**
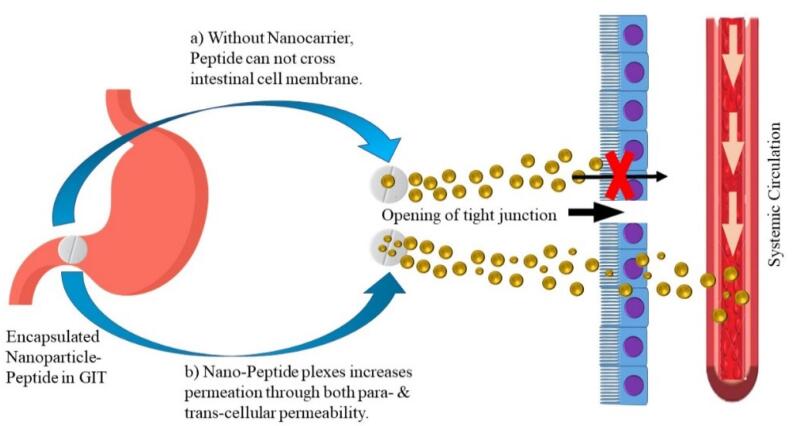
Nanoparticulate delivery of biologics to enhance absorption

 Nanotechnology has been proven to bridge the gap between physical and biological sciences by employing nanostructures and nanophases in a variety of disciplines of research, particularly in nanomedicine and nano-based medication delivery systems. NPs may be utilized as therapeutic molecule delivery systems, which can be accomplished in a variety of ways, including either by dissolving the protein and peptides in the carrier system, or by encapsulation mechanism i.e. encapsulating the peptide molecules in the nanoscale particle, and lastly by absorbing the bioactive agents onto the NP surface.^156^ Protection from acid and proteolytic enzymes in the GIT; delayed, controlled, target release of active molecule; mucus layer penetrability due to their nano size; large surface for the reactivity to the mucosal and various other layers/membranes; and ability to deliver cargo via the oral route for improved absorption. As seen in Figure 5, these characteristics enable NPs to improve biologic absorption. NPs with numerous functional characteristics aid in transport of variety of macromolecules like antibodies and active polypeptides to the immune system.^157,158^

 Insulin is one therapeutic peptide which has been extensively investigated for oral administration especially using NPs. Insulin when encapsulated inside NPs has been demonstrated to be less sensitive to enzymatic degradation and better absorbed due to the association with polymers. Moreover, the submicron size of the NP favours absorption through the intestinal membranes.^159,160^ In another study that investigated particle size, insulin NPs of diameter 345 nm showed better hypoglycemic effect than 123 nm particles in diabetic rats.^161^ This study also reported that the polymeric material chitosan could adhere to the intestinal membranes and transiently open the tight junctions. Chitosan and its derivatives are relatively non-toxic and biocompatible. Therefore, they have been widely studied as polymeric matrix for NP-mediated oral delivery of insulin. Chen et al^162^ developed multifunctional insulin NPs with dicyandiamide-modified chitosan, octaarginine, and hydrophilic hyaluronic acid (HA). The modified chitosan demonstrated improved water solubility, thereby forming NPs in neutral conditions. HA was employed for mucus penetrability and facilitating carrier interactions with the intestinal wall.

 NP absorption is mostly mediated through Peyer’s patches, particularly in the ileum following oral administration. They may be transported in between the cells and reach the jejunum. NPs can travel through the cellular spaces produced at the villi tip by the shedding of absorptive cells.^163^ For the first time, a recent study shed light on the impact of numerous variables on the release behaviour of protein from nanospheres.^164^ Protein-loaded (freeze-dried) nanospheres resulted in large burst release of protein, whereas non-freeze dried nanospheres showed minor release of model drug. Non-lyophilized nanospheres, on the other hand, revealed a rather quick release of protein till the succeeding month. However, because the NPs concentrate in the liver, one drawback of this type of delivery method is liver damage. Furthermore, particle size is restricted to 5–20 nm in diameter, making macromolecular loading a difficult procedure.

####  Liposomes

 Nanostructured lipid-based carrier systems, such as liposomes, nanoemulsions, solid lipid nanocarriers (SLNs), and others, have significant advantages over conventional drug forms since they are made of bio-based, non-toxic, non-irritant lipids and have ability to influence drug absorption in the intestine. The most widely utilized lipid-based carriers are liposomes. In the GIT, however, they are unstable and have poor penetration through the mucosal membrane. Nevertheless, these issues can be significantly minimized by modifying the liposome surface with a mucoadhesive polymer and protease inhibitors. When a chitosan (mucoadhesive polymer)-aprotinin (protease inhibitor) conjugate was given orally, a 15-fold higher AUC (area under curve) was observed than calcitonin alone.^165^ Degim et al^166^ observed that insulin permeability through Caco-2 cell monolayers and rabbit nasal mucosa connected to a diffusion cell was increased (on treatment with insulin-loaded liposomes and presence of permeation enhancers). Oral formulations were administered, blood glucose levels were measured, and compared to the results of the Caco-2 cell experiment. Finally, when the liposome sodium taurocholate (NaTC) formulation was employed, insulin permeability was enhanced across the Caco-2 cell monolayer. Liposome stability problems and protein breakdown during protein synthesis due to the use of organic solvents are the main disadvantages of liposomal peptide and protein delivery.^167^ Additionally, presence of bile may solvate the liposomes, causing them to burst and release the enclosed therapeutic macromolecules into the gut and their *in vivo* stability is viewed as a concern with their usage as non-invasive delivery methods.

####  Micelles

 Micelles are comprised of amphiphilic molecules that self-assemble. Micelles are generally spherical with varying diameter (2 to 20 nm) based upon their configuration. The structures have a hydrophilic/polar (head) and a hydrophobic/nonpolar (tail) portion. Micelles are made in aqueous phase, with the hydrophilic group facing outward and lipophilic group constituting inner core of micelles.^168^ Micelles can deliver hydrophilic as well as lipophilic molecules. These molecules can transport macromolecules because they can provide prolonged and regulated release, physicochemical stability of the incorporated macromolecules, improved pharmacology of active substance, and favourable distribution of molecules in tissues, and leading to enhance the absorption and bioavailability of therapeutics.^169^ Liposomes may have difficulties reaching the target location of action due to their large size than the vascular cut-off size in some cancers. Micelles may be a better option if this is the case. Hydrophilic–hydrophobic block copolymers can also be seen in polymeric micelles. Li et al^170^ investigated on functionalized polymeric micelles composed of folate conjugated bovine serum albumin (FA-BSA) and packed with super paramagnetic iron oxide nanoparticles (SPIONS). These polymeric micelles are used to target tumours and perform magnetic resonance imaging. In vitro investigations on folate receptor positive hepatoma cells revealed a higher cellular uptake. *In vivo* results revealed the potential of FA-BSA modified magnetic micelles as a tumor-targeting MRI probe. Researchers have found encouraging findings in oral administration of compound, which penetrate the gut membrane. Polymeric micelles can therefore be used to deliver macromolecules orally.^171^ However, micelles have intrinsic issues that might prohibit them from being utilised in therapeutic protein delivery, such as limited drug loading capacity, low water stability, poor half-life, significant toxicity and others.^172^

####  Microspheres

 Microspheres are spherical particles that range in size from 1 to 1000 mm. Natural or synthetic materials can be used to make microparticles. Lactoglobulin PLGA microspheres were given orally to neonates who were allergic to milk proteins. This formulation included Tween 20, which improved protein encapsulation efficiency and regulated release.^173^

 Insulin-loaded microparticles were made utilising a water-in-oil-in-oil emulsion method with a dispersed phase of PLG/PEG or PLA/PEG dissolved in dichloromethane and a continuous phase of 10% PVP-methanol. *In vitro* release characteristics of encapsulated insulin revealed a sustained-release property for a month. The usage of blended microparticles contributed in elevated insulin administration efficiency and consistent release over four weeks, as well as enhanced insulin stability.^174^

 Chemically modified soybean hydrolysate containing aromatic acyl chlorides can also be used to make stable pH-sensitive microspheres. These low-cost microspheres were soluble at pH values greater than 5.0 and stable at acid pH ( < 3.5).^175^ From a microsphere-hydrogel drug delivery system, Osswald and Kang-Mieler^176^ studied controlled and prolonged release of model agents. Bioactive anti-vascular endothelial growth factor (VEGF) drugs (ranibizumab or aflibercept) were employed as the model agents in the study. The drug delivery system can release either of anti-VEGF for up to 200 days. In human umbilical vascular endothelial cells, release samples revealed to inhibited proliferation of cells and no harm at any time.

 Increased medicament loading and prolonged delivery have been seen in several of these preparations. Microparticles can contain biologics with therapeutic activity to treat a variety of illnesses, including ophthalmology, cancer, heart problems, and inflammation.

###  Site-specific delivery: GIT

 Several factors involved in delivering macromolecules vary across the various areas of gut. Difference in absorption may be because of different pH values at GIT sites, as well as different level of proteolytic enzymes present in gut wall. Solubility and stability of PPs are affected by pH ranges and the degradation rate of the same is influenced by proteolytic enzymes.^177^ Depending upon the absorption location, the rate of absorption of peptides varies. As a result, numerous efforts to find the optimum absorption location in the gut have been made. Peptides have been released at a specific region of the GIT where entry into the lymphatic vessels is highest or biocatalyst actions are lowest to improve drug absorption after oral administration.

 Drug transport to the colon offers numerous advantages, including a longer residence period, lower enzymatic activity, and greater tissue response to absorption enhancers.^178^ For site-specific drug delivery, many techniques have been used, including magnetic systems, and mucoadhesive systems. Prodrug approach, azo-polymeric pro drug technique, pH-modulated, microbially triggered delivery systems, time-modulated system and pressure dependant release systems have all been tried for colon-specific administration, but with limited results.^179^ Developing pH-sensitive delivery systems that release loaded components according to pH of the surrounding medium. They can be influenced by the presence of food as well as a serious illness in the GIT that causes pH alterations.

 As a result, methods that rely on enzyme-controlled therapeutic peptide molecule release are more promising in this respect.^180^ Several *in vitro* studies revealed that approaches like penetration enhancers, or along with mucoadhesive devices were used as promising agents for delivery of insulin via oral route. For buccal administration of pressurized spray dosage form containing, a combination of penetration enhancers and micelles has been designed and tested using metered-dose inhaler. Bioavailability of the system was limited, and more research is needed to show its effectiveness. Multi-layered epithelial mucosa and constant flow of saliva are limitations for oral-buccal peptide drug administration.^181^

###  Membrane transporters

 In epithelial cells, several kinds of membrane transporter proteins are expressed which helps in transit the macromolecules like polypeptides to aid absorption. However, such carriers might be extremely beneficial in designing and developing the oral delivery of biologics. The drug should have morphological resemblance to carry by these membrane transporters. Because of these similarities in the structure, the membrane transporters present in vivo will couple the biomolecule which aids in transiting across the biological layer to reach the blood stream. Peptide transporters 1 and 2 (PepT1 and PepT2) are two distinct proton-coupled oligopeptide transporters that play a role in the transfer of amino acids in peptide form. The most significant differences among these two carriers are substrate binding and selectivity, as well as transportation capacity.^182^ For di- and tri-peptides, PepT1 works as a low-affinity/high-capacity transporter present mostly in the gut, has not been found in BBB and parenchyma, whereas PepT2 acts as a high-affinity/low-capacity transporter with a larger tissue distribution than Pept1, with the highest expression in the kidney. These peptide transporters are energy-dependent proton-coupled transporters. They may transport hydrophilic peptidomimetic medicines in contrast to their natural substrates (dipeptides and tripeptides).^182^

**Figure 6:**
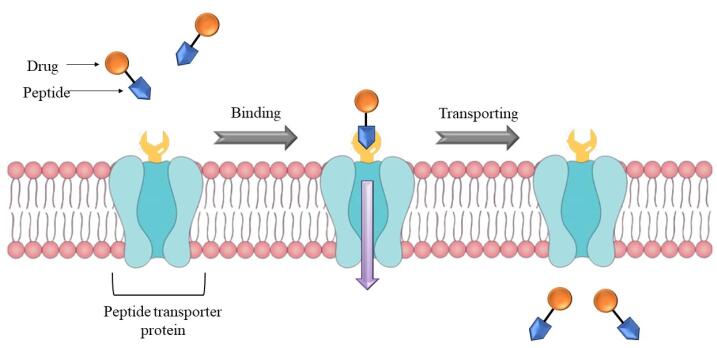
Usage of peptide transporter to deliver a peptide linked drug across the membrane

 The drug molecule is linked to a di/tri peptide already recognized as PepT1 transporter, and then, the entire molecule is transported through the epithelium (Figure 6). The critical parameter for peptide to couple with these transporters is its enzymatic stability, or else the peptide will enzymatically hydrolyse before it reaches the transporter which may prevent the transporter from recognising the peptide, resulting in no transportation. Membrane transporter proteins can often only transport molecules that are quite compact. As aforementioned, molecules of a greater size are generally transported by receptor-mediated endocytosis. They are crucial for the oral absorption of therapeutics like β-lactam antibiotics, angiotensin-converting enzyme inhibitors, nucleoside/nucleotide reverse transcriptase inhibitors, and renin inhibitors.^183^

###  Novel approaches

####  Self-nanoemulsifying drug delivery systems (SNEDDS)

 SNEDDS are formulations which are a characteristic mixture of lipid phase (oils), surface active agents (surfactants), and co-surfactants molecules in a definite stable proportion. These systems may generate transparent nanoemulsions with a diameter of 20 to 200 nm.^184^ In comparison to other nanocarriers such as polymeric NPs, liposomes, SLN, niosomes, micelles, carbon nanotubes, SNEDDS have garnered a lot of interest in the last decade due to their ease of scale-up and cost-effective approach.^185^ Advancement in SNEDDS formulation have improved activity characteristics like increased GI transit time, acidic environment resistance, enhance mucus diffusion, boosts penetration and upgraded cellular absorption, resulting in greater oral bioavailability of encapsulated therapeutics.^186,187^

 The high surface area of the ultrafine droplets allows for quick intestinal permeability. Proteins are protected from aqueous hydrolysis by the anhydrous nature of SNEDDS. Other bioactive effects of SNEDDS, such as tight junction opening and increased lymphatic absorption, also contribute to loaded protein therapies’ increased oral bioavailability. SNEDDS have been used to deliver biologics in several investigations.^187^ To enhance insulin oral bioavailability, Bravo-Alfaro et al^188^ generated SNEDDS using insulin complex and phosphatidylcholine (modified or unmodified). SNEDDSs were given in *in vitro* GI environment, and upon reaching last step of simulated small intestine they demonstrated 35.7% bioavailability. In diabetic rats, 36.1% reduction in blood sugar level was found after 4 h of receiving the modified phospholipid SNEDDS. The subcutaneous insulin injection generated 161.5 ± 24.8 IU/mL, the greatest quantity in blood, according to bioavailability tests. SNEDDS formulation was investigated by Karamanidou et al^189^ employing insulin/dimyristoyl phosphatidylglycerol, which was shown to be an effective mucus penetration enhancer with a 70.89% entrapment efficiency. Intestinal enzymes (trypsin, -chymotrypsin) were shown to effectively shield the therapeutic protein from enzymatic breakdown. Increased mucus permeability was seen in the SNEDDS formulation, which did not appear to be influenced by ionic strength. The addition of insulin-dimyristoyl phosphatidylglycerol to SNEDDS prevented an early surge of insulin release. However, because of fully lipidic nature of SNEDDS, incorporating any hydrophilic molecule in the formulation is challenging.

####  Eligen technology

**Figure 7:**
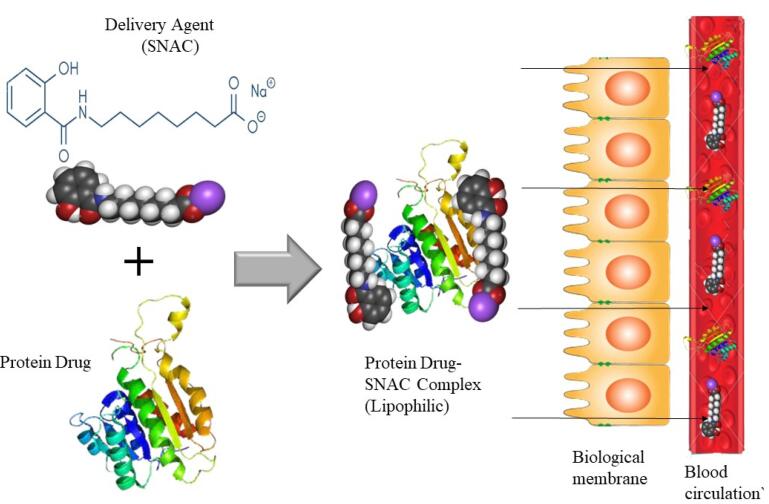
Emisphere Eligen® oral protein delivery technology

 The Eligen^®^ technology, which is used for oral administration, is based on the creation of unique delivery agents known as Emisphere delivery agents. It is a platform for delivering macromolecules that uses a macromolecule as an absorption enhancer. The macromolecule forms a weak, noncovalent bond with the drug molecules, allowing the medication to stay chemically unchanged. Eligen^®^ (Emisphere) is a drug delivery system that use sodium N-(8-(2-hydroxybenzoyl) amino caprylate) (SNAC) as a carrier molecule for weak non-covalently attaching medicines.^190^ Figure 7 describes the process. As per Emisphere, SNAC improves transcellular absorption without disrupting rigid connections. Prior to absorption, the process for proteins might entail a reversible change in protein structure and protection from destruction.^191^ SNAC increases insulin absorption transcellularly by a factor of ten without causing tight junction damage. It also protects the linked Protein /SNAC protein from proteolytic enzymes, which helps to keep it stable in the GI system.^192^

####  Peptelligence

 Peptelligence is a revolutionary oral medication technique for peptides and small compounds developed by Enteris Biopharma, USA. Peptides and other biopharmaceutical molecules classified as class II, III, or IV in the BCS may now be delivered orally as enteric-coated tablets using this cutting-edge technique.^192^ This technique was developed by solving two major challenges: solubility and permeability. The initial component of Peptelligence technology is a absorption enhancer, helps in penetrating the molecule by disturbing the rigid barrier in enterocytes and leading to transportation of macromolecules between the cells.^193^ A surfactant, which improves permeability, also works as a powerful solubilizer. Citric acid, the other primary excipient, is a calcium chelator and membrane penetration enhancer, as well as a pH-lowering agent that enhances absorptive flux and a membrane wetting/charge dispersion agent. Peptelligence has developed salmon calcitonin, leuprolide, and Ovarest, which were in phase II studies. Sodium caprylate plays a critical role in permeability enhancement, allowing molecules to pass across paracellular tight junctions in a temporary and reversible manner. In a phase III clinical trial, the oral delivery of octreotide was given to the patients with acromegaly. The formulation was examined for its single therapy potential and proven to be effective and safe in human subjects. To conclude, octreotide along with permeation enhancer showed same drug concentration profile as that produced by octreotide injection.^194^

####  Self-assembling bubble carrier approach

 By packing insulin as a protein drug in an enteric coated gelatin capsule, Lin and coworkers^195^ proposed developing a bubble carrier method. The insulin molecule is additionally shielded from protease inhibitors, and permeability is improved due to the addition of an absorption enhancer. The goal was to administer the insulin via oral route, which had the potential to be used for the oral administration of other pharmaceutical macromolecules as well. This self-assembling bubble carrier was encased in gelatin network and designed to release in intestine. When the designed dosage form outreaches lower intestinal secretions, it degrades and releases acid and alkali ions, which swiftly release carbon dioxide, that serves as transporter for protein insulin. In diabetic rats, the SDS-containing bubble carrier technology enhanced oral absorption of insulin, which had a significant blood glucose-lowering impact. However, employing SDS at the dosages used in this investigation had no effect on epithelial cell structural integrity, and there was no evidence of LPS absorption into the systemic circulation. The bubble carrier system with SDS provides a powerful and safe delivery method for oral insulin administration, according to these findings. As a result, the self-assemble bubble carrier system acts as the effective carrier and improves absorption.^196^

####  Without delivery technology 

 The development of oral formulations for biologic medicines in adults has received a lot of attention. This is pertinent in the case of infants and neonates who may receive vaccines, nutrients, and for whom oral delivery is an attractive option. Whitehead and team determined the viability of delivering peptide and protein medicines orally without the use of permeation enhancers or other aids. They tested their theory using the non-everted gut sac technique and discovered that macromolecular (FITC-Dextran) permeability was affected by molecule size, animal age, and tissue type. According to the findings, paracellular route in infant mice is more permeable than in adult mouse intestinal tissue. Even with a macromolecule as large as 70 kDa Dextran, infant small intestine tissue was substantially more permeable than adult tissue. This prompted them to investigate the oral absorption of both a moderately sized peptide (insulin) and a large protein (lactoferrin) with surprising results in infant and adult mice.^197^

## Lymphatic targeting/ lymphatic absorption

 The lymphatic system is a vascular network that drains protein-rich lymph from tissues. The lymphatic system, unlike the blood vascular system, is a one-way transit channel from the extracellular space to the venous system. The lymph system demands special attention when it comes to deliver biologics and other structural analogues because of some special benefits. To begin, this aforementioned system and lymph nodes are attractive targets for cancer treatment, and in some metastatic tumours, lymph nodes are a viable option. Second, lymph nodes are home to thymus-dependent small and large lymphocytes, as well as macrophages, which generate circulation antibodies that trigger immunological responses.^198^ When macromolecules > 20 kDa in size or 10–100 nm (diameter) are administered via oral route and eventually reach into a lumen, they must first transit the epithelial membrane, before entering the lymphatic and capillary system. The transcellular lipid route and the paracellular pathway, which operates by adding absorption enhancers, are two methods via which the macromolecules migrate to lymphatic system. Transcytosis via Peyer’s patches is the second pathway, which appears to be most suited for very powerful chemicals like lymphokines and vaccinations. Small molecules can be stimulated to enter the lymphatic system in one of two ways: by administering them in conjunction with synthetic macromolecular constructions, or by hitchhiking on endogenous cells or macromolecular carriers that are carried from tissues to the lymphatics.^199^ Deak and Csáky^200^ performed a series of studies in normal and cirrhotic rats to investigate the variables that govern the absorption of compounds (supplements, drug) from intestinal space into the lymphatic system. Intestinal lymph, portal venous plasma, and intestinal perfusate were all evaluated simultaneously after test chemicals were administered through in situ jejunal luminal perfusion or systemic intravenous infusion. Some of the variables impacting macromolecule absorption, such as the permeability of the compound into the intestinal lymphatic system and lipophilicity, came into play. They concluded that molecule size has a major impact on relative distribution. Because if particles are too big to pass through the fenestrated blood capillaries of the mucosa, they must be carried via lymph. Chylomicron is a good example. The use of formulations containing a long chain and unsaturated fatty acid, as well as a surfactant, promotes the transfer of biologics into lymph.^201^ The physicochemical properties of smaller molecules (peptides) are one of the most important variables in lymphatic absorption. Highly lipophilic substances facilitate lymphatic flow.

 The design of formulation which precisely gunshots the lymphatic system is a second option. Bleomycin, an anticancer drug, was used in one such delivery method. Bleomycin was discovered in significant quantities in the lymph after treatment with absorption enhancer as unsaturated fatty acids.^202^ The M-cell pathway looks to be extremely promising for oral administration of biologics to local lymph nodes using carrier systems.

## Future trends

 The oral route is popular since it is straightforward and is low-cost. In the GIT, PPs are easily digested, and the hydrophilic nature of most endogenous peptides prevents them from crossing the epithelial barrier. Because of the complicated structure and diversity of PPs, no universally applicable strategy to deliver biologics were identified; several solutions inside this review specifically tailored for the peptide delivery are aforementioned. Several researchers have made efforts to promote absorption across the GIT in the development of new macromolecule technologies, and a number of these methods have shown promise in clinical studies. For PP drug delivery, PEGylation, mannosylation, glycosylation, cell-penetrating peptides (CPPs), targeted delivery, site-specific delivery, and mucoadhesive polymeric system are all potential possibilities. Researchers are currently combining approaches to improve oral bioavailability, such as CPPs with chitosan NPs, CPP with transcytosing peptides, delivery using polysaccharides as carriers, such as cellulose derivatives, alginates, cyclodextrin, CPP with PLA-NPs containing cyclic arginine, chitosan-taurocholic acid.

 Recently, some new techniques have been employed to overcome the barrier faced by oral bioavailability of biologics Eudratech Pep Technology, Peptelligence and Eligen Technology.

 Some new techniques for improving the bioavailability of oral peptides are under clinical investigation, such as the self-orienting millimeter scale applicator (SOMA), microneedles, luminal unfolding microneedle injector (LUMI), RaniPill, iontophoretic patch, use of sonophoresis technology, electroporation and iontophoresis techniques, micro-container with permeation enhancer, and peptidase and protease inhibitor.

## Challenges

 Oral delivery of therapeutic PPs is still not as straightforward as delivery of small molecules. There are formulation challenges, an increased number of manufacturing steps, control on environmental conditions during manufacture, packing, storage and distribution.

## Conclusion

 The increasing relevance of biologics as treatments in diversity of ailments has reignited interest in developing oral delivery systems for these agents. The therapeutic potential of orally administered proteins and peptides is limited by their systemic instability in the GIT, enzymatic and acid catalyzed breakdown, inappropriate lipophilicity, structural variations, and low membrane permeability, as well as inadequate absorption via intestinal epithelia. Because of the above-mentioned thorns, the pathway of improved absorption of macromolecules by peroral administration are still challenging. Significant attempts have been undertaken in recent years to improve the bioavailability of orally given macromolecules. To deal with the harsh environment of the GIT, systemic instability, and boost bioavailability, absorption enhancers, enzyme inhibitors, chemical modifications, particle delivery, lipid-based carrier, site specific delivery, polymeric conjugation, and carrier systems were developed. These techniques had some early success, but only a handful have made it to the clinics. Patients, healthcare professionals, and pharmaceutical manufacturers all have different needs, and an ideal oral formulation must fulfil all of them. The focus of the study has shifted to innovative and vibrant techniques such as Emisphere, SNEDDS, Bubble-carrier strategy and many more.

 Currently, researchers using combinational approaches for enhancing oral bioavailability like, cell penetrating peptides CPP with chitosan NPs, CPP with transcytosing peptides, delivery using polysaccharides as carriers such as cellulose derivative, alginates, cyclodextrin, CPP with PLA-NPs containing cyclic arginine, chitosan-taurocholic acid conjugates, folate mediated lipid NPs, PEGylated lipid nanocapsules achieved by targeted NPs. As the significance of proteins and peptides in increasing along with their market share in pharmaceuticals, we believe that this review will guide the formulation scientist in selecting the optimal strategy for enhancing the therapeutic outcomes from their product.

## Competing Interests

 The authors declare that they have no conflicts of interest.

## Ethical Approval

 Not applicable.

## Funding

 None.

## References

[1] Makurvet FD (2021). Biologics vs small molecules: drug costs and patient access. Med Drug Discov.

[2] Mantaj J, Vllasaliu D (2020). Recent advances in the oral delivery of biologics. Pharm J.

[3] Watt PM (2006). Screening for peptide drugs from the natural repertoire of biodiverse protein folds. Nat Biotechnol.

[4] Watford M, Wu G (2018). Protein. Adv Nutr.

[5] Craik DJ, Fairlie DP, Liras S, Price D (2013). The future of peptide-based drugs. Chem Biol Drug Des.

[6] Kulkarni SS, Sayers J, Premdjee B, Payne RJ (2018). Rapid and efficient protein synthesis through expansion of the native chemical ligation concept. Nat Rev Chem.

[7] Jitendra Jitendra, Sharma PK, Bansal S, Banik A (2011). Noninvasive routes of proteins and peptides drug delivery. Indian J Pharm Sci.

[8] Chan J, Cheng-Lai A (2017). Inhaled insulin: a clinical and historical review. Cardiol Rev.

[9] Sousa F, Castro P, Fonte P, Sarmento B (2015). How to overcome the limitations of current insulin administration with new non-invasive delivery systems. Ther Deliv.

[10] Barua S, Kim H, Jo K, Seo CW, Park TJ, Lee KB (2016). Drug delivery techniques for buccal route: formulation strategies and recent advances in dosage form design. J Pharm Investig.

[11] Eek D, Krohe M, Mazar I, Horsfield A, Pompilus F, Friebe R (2016). Patient-reported preferences for oral versus intravenous administration for the treatment of cancer: a review of the literature. Patient Prefer Adherence.

[12] Pridgen EM, Alexis F, Farokhzad OC (2015). Polymeric nanoparticle drug delivery technologies for oral delivery applications. Expert Opin Drug Deliv.

[13] Bruno BJ, Miller GD, Lim CS (2013). Basics and recent advances in peptide and protein drug delivery. Ther Deliv.

[14] Higashino H, Hasegawa T, Yamamoto M, Matsui R, Masaoka Y, Kataoka M (2014). In vitro-in vivo correlation of the effect of supersaturation on the intestinal absorption of BCS class 2 drugs. Mol Pharm.

[15] Jung T, Kamm W, Breitenbach A, Kaiserling E, Xiao JX, Kissel T (2000). Biodegradable nanoparticles for oral delivery of peptides: is there a role for polymers to affect mucosal uptake?. Eur J Pharm Biopharm.

[16] Lambkin I, Pinilla C (2002). Targeting approaches to oral drug delivery. Expert Opin Biol Ther.

[17] des Rieux A, Fievez V, Garinot M, Schneider YJ, Préat V (2006). Nanoparticles as potential oral delivery systems of proteins and vaccines: a mechanistic approach. J Control Release.

[18] Lowman AM, Morishita M, Kajita M, Nagai T, Peppas NA (1999). Oral delivery of insulin using pH-responsive complexation gels. J Pharm Sci.

[19] Schilling RJ, Mitra AK (1991). Degradation of insulin by trypsin and alpha-chymotrypsin. Pharm Res.

[20] Werle M, Bernkop-Schnürch A (2006). Strategies to improve plasma half life time of peptide and protein drugs. Amino Acids.

[21] Langguth P, Merkle HP, Amidon GL (1994). Oral absorption of peptides: the effect of absorption site and enzyme inhibition on the systemic availability of metkephamid. Pharm Res.

[22] George M, Abraham TE (2006). Polyionic hydrocolloids for the intestinal delivery of protein drugs: alginate and chitosan--a review. J Control Release.

[23] Narayanan D, Anitha A, Jayakumar R, Chennazhi KP (2013). In vitro and in vivo evaluation of osteoporosis therapeutic peptide PTH 1-34 loaded pegylated chitosan nanoparticles. Mol Pharm.

[24] Anilkumar P, Badarinath AV, Naveen N, Prasad K, Reddy BR, Hyndhavi M (2011). A rationalized description on study of intestinal barrier, drug permeability and permeation enhancers. J Glob Trends Pharm Sci.

[25] Liu H, Meagher CK, Moore CP, Phillips TE (2005). M cells in the follicle-associated epithelium of the rabbit conjunctiva preferentially bind and translocate latex beads. Invest Ophthalmol Vis Sci.

[26] Greenwood-Van Meerveld B, Johnson AC, Grundy D (2017). Gastrointestinal physiology and function. Handb Exp Pharmacol.

[27] Evans DF, Pye G, Bramley R, Clark AG, Dyson TJ, Hardcastle JD (1988). Measurement of gastrointestinal pH profiles in normal ambulant human subjects. Gut.

[28] Koziolek M, Grimm M, Becker D, Iordanov V, Zou H, Shimizu J (2015). Investigation of pH and temperature profiles in the GI tract of fasted human subjects using the Intellicap® system. J Pharm Sci.

[29] Cheng H, Leblond CP (1974). Origin, differentiation and renewal of the four main epithelial cell types in the mouse small intestine V Unitarian theory of the origin of the four epithelial cell types. Am J Anat.

[30] Gebert A, Rothkötter HJ, Pabst R (1996). M cells in Peyer’s patches of the intestine. Int Rev Cytol.

[31] Kiela PR, Ghishan FK (2016). Physiology of intestinal absorption and secretion. Best Pract Res Clin Gastroenterol.

[32] Stillhart C, Vučićević K, Augustijns P, Basit AW, Batchelor H, Flanagan TR (2020). Impact of gastrointestinal physiology on drug absorption in special populations--an UNGAP review. Eur J Pharm Sci.

[33] Carino GP, Mathiowitz E (1999). Oral insulin delivery. Adv Drug Deliv Rev.

[34] Patel G, Misra A. Oral delivery of proteins and peptides: concepts and applications. In: Misra A, ed. Challenges in Delivery of Therapeutic Genomics and Proteomics. Amsterdam, NL: Elsevier; 2011. p. 481-529. 10.1016/b978-0-12-384964-9.00010-4.

[35] Edelblum KL, Turner JR. Epithelial cells: structure, transport, and barrier function. In: Mestecky J, Strober W, Russell MW, Kelsall BL, Cheroutre H, Lambrecht BN, eds. Mucosal Immunology. 4th ed. Amsterdam, NL: Academic Press; 2015. p. 187-210. 10.1016/b978-0-12-415847-4.00012-4.

[36] Farquhar MG, Palade GE (1963). Junctional complexes in various epithelia. J Cell Biol.

[37] Tash BR, Bewley MC, Russo M, Keil JM, Griffin KA, Sundstrom JM (2012). The occludin and ZO-1 complex, defined by small angle X-ray scattering and NMR, has implications for modulating tight junction permeability. Proc Natl Acad Sci U S A.

[38] Alagga AA, Gupta V. Drug absorption. In: StatPearls [Internet]. Treasure Island, FL: StatPearls Publishing; 2022. Available from: https://www.ncbi.nlm.nih.gov/books/NBK557405/.

[39] Conradi RA, Hilgers AR, Ho NF, Burton PS (1991). The influence of peptide structure on transport across Caco-2 cells. Pharm Res.

[40] Mandracchia D, Rosato A, Trapani A, Chlapanidas T, Montagner IM, Perteghella S (2017). Design, synthesis and evaluation of biotin decorated inulin-based polymeric micelles as long-circulating nanocarriers for targeted drug delivery. Nanomedicine.

[41] Jain D, Raturi R, Jain V, Bansal P, Singh R (2011). Recent technologies in pulsatile drug delivery systems. Biomatter.

[42] Giannasca PJ, Giannasca KT, Leichtner AM, Neutra MR (1999). Human intestinal M cells display the sialyl Lewis A antigen. Infect Immun.

[43] Brayden DJ, Jepson MA, Baird AW (2005). Keynote review: intestinal Peyer’s patch M cells and oral vaccine targeting. Drug Discov Today.

[44] Clark MA, Hirst BH, Jepson MA (2000). Lectin-mediated mucosal delivery of drugs and microparticles. Adv Drug Deliv Rev.

[45] Kou L, Sun J, Zhai Y, He Z (2013). The endocytosis and intracellular fate of nanomedicines: implication for rational design. Asian J Pharm Sci.

[46] Shima H, Watanabe T, Fukuda S, Fukuoka S, Ohara O, Ohno H (2014). A novel mucosal vaccine targeting Peyer’s patch M cells induces protective antigen-specific IgA responses. Int Immunol.

[47] Snoeck V, Goddeeris B, Cox E (2005). The role of enterocytes in the intestinal barrier function and antigen uptake. Microbes Infect.

[48] Barthe L, Woodley J, Houin G (1999). Gastrointestinal absorption of drugs: methods and studies. Fundam Clin Pharmacol.

[49] Alsford S, Field MC, Horn D (2013). Receptor-mediated endocytosis for drug delivery in African trypanosomes: fulfilling Paul Ehrlich’s vision of chemotherapy. Trends Parasitol.

[50] Nighot PK, Leung L, Ma TY (2017). Chloride channel ClC- 2 enhances intestinal epithelial tight junction barrier function via regulation of caveolin-1 and caveolar trafficking of occludin. Exp Cell Res.

[51] Gabor F, Bogner E, Weissenboeck A, Wirth M (2004). The lectin-cell interaction and its implications to intestinal lectin-mediated drug delivery. Adv Drug Deliv Rev.

[52] Zhu Q, Chen Z, Paul PK, Lu Y, Wu W, Qi J (2021). Oral delivery of proteins and peptides: challenges, status quo and future perspectives. Acta Pharm Sin B.

[53] Brooks FP (1985). Effect of diet on gastric secretion. Am J Clin Nutr.

[54] Lennard-Jones JE, Fletcher J, Shaw DG (1968). Effect of different foods on the acidity of the gastric contents in patients with duodental ulcer 3 Effect of altering the proportions of protein and carbohydrate. Gut.

[55] Lennard-Jones JE, Babouris N (1965). Effect of different foods on the acidity of the gastric contents in patients with duodenal ulcer I A comparison between two ‘therapeutic’ diets and freely-chosen meals. Gut.

[56] Khan MS, Roberts MS (2018). Challenges and innovations of drug delivery in older age. Adv Drug Deliv Rev.

[57] Mooij MG, de Koning BA, Huijsman ML, de Wildt SN (2012). Ontogeny of oral drug absorption processes in children. Expert Opin Drug Metab Toxicol.

[58] Bai JPF, Burckart GJ, Mulberg AE (2016). Literature review of gastrointestinal physiology in the elderly, in pediatric patients, and in patients with gastrointestinal diseases. J Pharm Sci.

[59] Fallingborg J, Christensen LA, Jacobsen BA, Rasmussen SN (1993). Very low intraluminal colonic pH in patients with active ulcerative colitis. Dig Dis Sci.

[60] Press AG, Hauptmann IA, Hauptmann L, Fuchs B, Fuchs M, Ewe K (1998). Gastrointestinal pH profiles in patients with inflammatory bowel disease. Aliment Pharmacol Ther.

[61] Hua S, Marks E, Schneider JJ, Keely S (2015). Advances in oral nano-delivery systems for colon targeted drug delivery in inflammatory bowel disease: selective targeting to diseased versus healthy tissue. Nanomedicine.

[62] Wang W (1999). Instability, stabilization, and formulation of liquid protein pharmaceuticals. Int J Pharm.

[63] Roxas M (2008). The role of enzyme supplementation in digestive disorders. Altern Med Rev.

[64] Dhillon A, Sharma K, Rajulapati V, Goyal A. Proteolytic enzymes. In: Pandey A, Negi S, Soccol CR, eds. Current Developments in Biotechnology and Bioengineering. Amsterdam, NL: Elsevier; 2017. p. 149-73. 10.1016/b978-0-444-63662-1.00007-5.

[65] Sanchón J, Fernández-Tomé S, Miralles B, Hernández-Ledesma B, Tomé D, Gaudichon C (2018). Protein degradation and peptide release from milk proteins in human jejunum Comparison with in vitro gastrointestinal simulation. Food Chem.

[66] Wang J, Yadav V, Smart AL, Tajiri S, Basit AW (2015). Toward oral delivery of biopharmaceuticals: an assessment of the gastrointestinal stability of 17 peptide drugs. Mol Pharm.

[67] Kim YS, Birtwhistle W, Kim YW (1972). Peptide hydrolases in the bruch border and soluble fractions of small intestinal mucosa of rat and man. J Clin Invest.

[68] Woodley JF (1994). Enzymatic barriers for GI peptide and protein delivery. Crit Rev Ther Drug Carrier Syst.

[69] Yatsunenko T, Rey FE, Manary MJ, Trehan I, Dominguez-Bello MG, Contreras M (2012). Human gut microbiome viewed across age and geography. Nature.

[70] Boegh M, Nielsen HM (2015). Mucus as a barrier to drug delivery – understanding and mimicking the barrier properties. Basic Clin Pharmacol Toxicol.

[71] Shan W, Zhu X, Liu M, Li L, Zhong J, Sun W (2015). Overcoming the diffusion barrier of mucus and absorption barrier of epithelium by self-assembled nanoparticles for oral delivery of insulin. ACS Nano.

[72] Wright L, Barnes TJ, Prestidge CA (2020). Oral delivery of protein-based therapeutics: gastroprotective strategies, physiological barriers and in vitro permeability prediction. Int J Pharm.

[73] Bansil R, Turner BS (2018). The biology of mucus: composition, synthesis and organization. Adv Drug Deliv Rev.

[74] Larhed AW, Artursson P, Gråsjö J, Björk E (1997). Diffusion of drugs in native and purified gastrointestinal mucus. J Pharm Sci.

[75] Sinko PJ, Ming H, Amidon GL (1987). Carrier mediated transport of amino acids, small peptides, and their drug analogs. J Control Release.

[76] Maher S, Mrsny RJ, Brayden DJ (2016). Intestinal permeation enhancers for oral peptide delivery. Adv Drug Deliv Rev.

[77] Homayun B, Lin X, Choi HJ (2019). Challenges and recent progress in oral drug delivery systems for biopharmaceuticals. Pharmaceutics.

[78] Banerjee A, Qi J, Gogoi R, Wong J, Mitragotri S (2016). Role of nanoparticle size, shape and surface chemistry in oral drug delivery. J Control Release.

[79] Liu C, Kou Y, Zhang X, Cheng H, Chen X, Mao S (2018). Strategies and industrial perspectives to improve oral absorption of biological macromolecules. Expert Opin Drug Deliv.

[80] Qi J, Zhuang J, Lv Y, Lu Y, Wu W (2018). Exploiting or overcoming the dome trap for enhanced oral immunization and drug delivery. J Control Release.

[81] Du D, Wang-Kan X, Neuberger A, van Veen HW, Pos KM, Piddock LJV (2018). Multidrug efflux pumps: structure, function and regulation. Nat Rev Microbiol.

[82] Bruckmueller H, Martin P, Kähler M, Haenisch S, Ostrowski M, Drozdzik M (2017). Clinically relevant multidrug transporters are regulated by microRNAs along the human intestine. Mol Pharm.

[83] Matsson P, Kihlberg J (2017). How big is too big for cell permeability?. J Med Chem.

[84] Donovan MD, Flynn GL, Amidon GL (1990). Absorption of polyethylene glycols 600 through 2000: the molecular weight dependence of gastrointestinal and nasal absorption. Pharm Res.

[85] Matsuyama T, Morita T, Horikiri Y, Yamahara H, Yoshino H (2006). Enhancement of nasal absorption of large molecular weight compounds by combination of mucolytic agent and nonionic surfactant. J Control Release.

[86] Santos GB, Ganesan A, Emery FS (2016). Oral administration of peptide-based drugs: beyond Lipinski’s rule. ChemMedChem.

[87] Tavakoli-Saberi MR, Audus KL (1989). Cultured buccal epithelium: an in vitro model derived from the hamster pouch for studying drug transport and metabolism. Pharm Res.

[88] Wang B, Xie N, Li B (2019). Influence of peptide characteristics on their stability, intestinal transport, and in vitro bioavailability: a review. J Food Biochem.

[89] Liaw J, Rojanasakul Y, Robinson JR (1992). The effect of drug charge type and charge density on corneal transport. Int J Pharm.

[90] Rojanasakul Y, Wang LY, Bhat M, Glover DD, Malanga CJ, Ma JK (1992). The transport barrier of epithelia: a comparative study on membrane permeability and charge selectivity in the rabbit. Pharm Res.

[91] Merkle HP, Wolany G (1992). Buccal delivery for peptide drugs. J Control Release.

[92] Bak A, Leung D, Barrett SE, Forster S, Minnihan EC, Leithead AW (2015). Physicochemical and formulation developability assessment for therapeutic peptide delivery--a primer. AAPS J.

[93] Amidon GL, Lennernäs H, Shah VP, Crison JR (1995). A theoretical basis for a biopharmaceutic drug classification: the correlation of in vitro drug product dissolution and in vivo bioavailability. Pharm Res.

[94] Ferreira V, Velloso MI, Vita M, Landoni MF (2019). Vía intranasal: una alternativa para la administración de fármacos de acción central en equinos. Analecta Vet.

[95] Corbo DC, Liu JC, Chien YW (1989). Drug absorption through mucosal membranes: effect of mucosal route and penetrant hydrophilicity. Pharm Res.

[96] Alex A, Millan DS, Perez M, Wakenhut F, Whitlock GA (2011). Intramolecular hydrogen bonding to improve membrane permeability and absorption in beyond rule of five chemical space. Medchemcomm.

[97] Lipinski CA (2016). Rule of five in 2015 and beyond: target and ligand structural limitations, ligand chemistry structure and drug discovery project decisions. Adv Drug Deliv Rev.

[98] Wang W (2015). Advanced protein formulations. Protein Sci.

[99] Zapadka KL, Becher FJ, Gomes Dos Santos AL, Jackson SE (2017). Factors affecting the physical stability (aggregation) of peptide therapeutics. Interface Focus.

[100] Williams AC, Barry BW (2004). Penetration enhancers. Adv Drug Deliv Rev.

[101] Maher S, Leonard TW, Jacobsen J, Brayden DJ (2009). Safety and efficacy of sodium caprate in promoting oral drug absorption: from in vitro to the clinic. Adv Drug Deliv Rev.

[102] Hamman JH, Enslin GM, Kotzé AF (2005). Oral delivery of peptide drugs: barriers and developments. BioDrugs.

[103] Lindmark T, Schipper N, Lazorová L, de Boer AG, Artursson P (1998). Absorption enhancement in intestinal epithelial Caco-2 monolayers by sodium caprate: assessment of molecular weight dependence and demonstration of transport routes. J Drug Target.

[104] Sawada T, Ogawa T, Tomita M, Hayashi M, Awazu S (1991). Role of paracellular pathway in nonelectrolyte permeation across rat colon epithelium enhanced by sodium caprate and sodium caprylate. Pharm Res.

[105] Bies C, Lehr CM, Woodley JF (2004). Lectin-mediated drug targeting: history and applications. Adv Drug Deliv Rev.

[106] Anderberg EK, Nyström C, Artursson P (1992). Epithelial transport of drugs in cell culture VII: effects of pharmaceutical surfactant excipients and bile acids on transepithelial permeability in monolayers of human intestinal epithelial (Caco-2) cells. J Pharm Sci.

[107] Yang T, Arnold JJ, Ahsan F (2005). Tetradecylmaltoside (TDM) enhances in vitro and in vivo intestinal absorption of enoxaparin, a low molecular weight heparin. J Drug Target.

[108] Raiman J, Törmälehto S, Yritys K, Junginger HE, Mönkkönen J (2003). Effects of various absorption enhancers on transport of clodronate through Caco-2 cells. Int J Pharm.

[109] Yeh PY, Berenson MM, Samowitz WS, Kopečková P, Kopecek J (1995). Site-specific drug delivery and penetration enhancement in the gastrointestinal tract. J Control Release.

[110] Cox DS, Raje S, Gao H, Salama NN, Eddington ND (2002). Enhanced permeability of molecular weight markers and poorly bioavailable compounds across Caco-2 cell monolayers using the absorption enhancer, zonula occludens toxin. Pharm Res.

[111] Bowe CL, Mokhtarzadeh L, Venkatesan P, Babu S, Axelrod HR, Sofia MJ (1997). Design of compounds that increase the absorption of polar molecules. Proc Natl Acad Sci U S A.

[112] Swenson ES, Milisen WB, Curatolo W (1994). Intestinal permeability enhancement: efficacy, acute local toxicity, and reversibility. Pharm Res.

[113] Borchard G, Lueβen HL, de Boer AG, Verhoef JC, Lehr CM, Junginger HE (1996). The potential of mucoadhesive polymers in enhancing intestinal peptide drug absorption III: effects of chitosan-glutamate and carbomer on epithelial tight junctions in vitro. J Control Release.

[114] Thanou M, Verhoef JC, Junginger HE (2001). Oral drug absorption enhancement by chitosan and its derivatives. Adv Drug Deliv Rev.

[115] Hochman J, Artursson P (1994). Mechanisms of absorption enhancement and tight junction regulation. J Control Release.

[116] Anderberg EK, Artursson P (1993). Epithelial transport of drugs in cell culture VIII: effects of sodium dodecyl sulfate on cell membrane and tight junction permeability in human intestinal epithelial (Caco-2) cells. J Pharm Sci.

[117] Davis SS, Illum L (2003). Absorption enhancers for nasal drug delivery. Clin Pharmacokinet.

[118] Leonard TW, Lynch J, McKenna MJ, Brayden DJ (2006). Promoting absorption of drugs in humans using medium-chain fatty acid-based solid dosage forms: GIPET. Expert Opin Drug Deliv.

[119] Tuvia S, Pelled D, Marom K, Salama P, Levin-Arama M, Karmeli I (2014). A novel suspension formulation enhances intestinal absorption of macromolecules via transient and reversible transport mechanisms. Pharm Res.

[120] Verboni M, Perinelli DR, Qiu CY, Tiboni M, Aluigi A, Lucarini S (2023). Synthesis and properties of sucrose- and lactose-based aromatic ester surfactants as potential drugs permeability enhancers. Pharmaceuticals (Basel).

[121] Fein KC, Gleeson JP, Cochran K, Lamson NG, Doerfler R, Melamed JR (2023). Long-term daily oral administration of intestinal permeation enhancers is safe and effective in mice. Bioeng Transl Med.

[122] Tyagi P, Trivedi R, Pechenov S, Patel C, Revell J, Wills S (2021). Targeted oral peptide delivery using multi-unit particulates: drug and permeation enhancer layering approach. J Control Release.

[123] Park K, Kwon IC, Park K (2011). Oral protein delivery: current status and future prospect. React Funct Polym.

[124] Bernkop-Schnürch A (1998). The use of inhibitory agents to overcome the enzymatic barrier to perorally administered therapeutic peptides and proteins. J Control Release.

[125] Wagner AM, Gran MP, Peppas NA (2018). Designing the new generation of intelligent biocompatible carriers for protein and peptide delivery. Acta Pharm Sin B.

[126] Friedman DI, Amidon GL (1991). Oral absorption of peptides: influence of pH and inhibitors on the intestinal hydrolysis of leu-enkephalin and analogues. Pharm Res.

[127] Baas TC, Thacker PA (1996). Impact of gastric pH on dietary enzyme activity and survivability in swine fed β-glucanase supplemented diets. Can J Anim Sci.

[128] Piper DW, Fenton BH (1965). pH stability and activity curves of pepsin with special reference to their clinical importance. Gut.

[129] Hyun HH, Zeikus JG (1985). General biochemical characterization of thermostable extracellular beta-amylase from Clostridium thermosulfurogenes. Appl Environ Microbiol.

[130] Renukuntla J, Vadlapudi AD, Patel A, Boddu SH, Mitra AK (2013). Approaches for enhancing oral bioavailability of peptides and proteins. Int J Pharm.

[131] Reseland JE, Holm H, Jacobsen MB, Jenssen TG, Hanssen LE (1996). Proteinase inhibitors induce selective stimulation of human trypsin and chymotrypsin secretion. J Nutr.

[132] Otsuki M, Ohki A, Okabayashi Y, Suehiro I, Baba S (1987). Effect of synthetic protease inhibitor camostate on pancreatic exocrine function in rats. Pancreas.

[133] Melmed RN, El-Aaser AA, Holt SJ (1976). Hypertrophy and hyperplasia of the neonatal rat exocrine pancreas induced by orally administered soybean trypsin inhibitor. Biochim Biophys Acta.

[134] Borchardt RT (1999). Optimizing oral absorption of peptides using prodrug strategies. J Control Release.

[135] Sandanaraj BS, Vutukuri DR, Simard JM, Klaikherd A, Hong R, Rotello VM (2005). Noncovalent modification of chymotrypsin surface using an amphiphilic polymer scaffold: implications in modulating protein function. J Am Chem Soc.

[136] Barbarić S, Leustek I, Pavlovic B, Cesi V, Mildner P (1988). Stabilization of glycoenzymes by cross-linking of their carbohydrate chains. Ann N Y Acad Sci.

[137] Pfister D, Morbidelli M (2014). Process for protein PEGylation. J Control Release.

[138] Goyon A, D’Atri V, Colas O, Fekete S, Beck A, Guillarme D (2017). Characterization of 30 therapeutic antibodies and related products by size exclusion chromatography: feasibility assessment for future mass spectrometry hyphenation. J Chromatogr B Analyt Technol Biomed Life Sci.

[139] Belletti D, Tosi G, Forni F, Lagreca I, Barozzi P, Pederzoli F (2016). PEGylated siRNA lipoplexes for silencing of BLIMP-1 in primary effusion lymphoma: in vitro evidences of antitumoral activity. Eur J Pharm Biopharm.

[140] Rahnama Yazdi J, Tafaghodi M, Sadri K, Mashreghi M, Nikpoor AR, Nikoofal-Sahlabadi S (2020). Folate targeted PEGylated liposomes for the oral delivery of insulin: in vitro and in vivo studies. Colloids Surf B Biointerfaces.

[141] Kerwin BA, Chang BS, Gegg CV, Gonnelli M, Li T, Strambini GB (2002). Interactions between PEG and type I soluble tumor necrosis factor receptor: modulation by pH and by PEGylation at the N terminus. Protein Sci.

[142] Zhang F, Liu MR, Wan HT (2014). Discussion about several potential drawbacks of PEGylated therapeutic proteins. Biol Pharm Bull.

[143] Dahan A, Khamis M, Agbaria R, Karaman R (2012). Targeted prodrugs in oral drug delivery: the modern molecular biopharmaceutical approach. Expert Opin Drug Deliv.

[144] Gangwar S, Pauletti GM, Wang B, Siahaan TJ, Stella VJ, Borchardt RT (1997). Prodrug strategies to enhance the intestinal absorption of peptides. Drug Discov Today.

[145] Tanaka K, Fujita T, Yamamoto Y, Murakami M, Yamamoto A, Muranishi S (1996). Enhancement of intestinal transport of thyrotropin-releasing hormone via a carrier-mediated transport system by chemical modification with lauric acid. Biochim Biophys Acta.

[146] Bundgaard H (1992). The utility of the prodrug approach to improve peptide absorption. J Control Release.

[147] Hu Y, Lin R, Zhang P, Fern J, Cheetham AG, Patel K (2016). Electrostatic-driven lamination and untwisting of β-sheet assemblies. ACS Nano.

[148] Ma W, Cheetham AG, Cui H (2016). Building nanostructures with drugs. Nano Today.

[149] Vagner J, Qu H, Hruby VJ (2008). Peptidomimetics, a synthetic tool of drug discovery. Curr Opin Chem Biol.

[150] Kharb R, Rana M, Sharma PC, Yar MS (2011). Therapeutic importance of peptidomimetics in medicinal chemistry. J Chem Pharm Res.

[151] Borchardt RT, Jeffrey A, Siahaan TJ, Gangwar S, Pauletti GM (1997). Improvement of oral peptide bioavailability: peptidomimetics and prodrug strategies. Adv Drug Deliv Rev.

[152] Abu-Qarn M, Eichler J, Sharon N (2008). Not just for Eukarya anymore: protein glycosylation in bacteria and archaea. Curr Opin Struct Biol.

[153] Lehle L, Strahl S, Tanner W (2006). Protein glycosylation, conserved from yeast to man: a model organism helps elucidate congenital human diseases. Angew Chem Int Ed Engl.

[154] Marth JD, Grewal PK (2008). Mammalian glycosylation in immunity. Nat Rev Immunol.

[155] Rajan RS, Li T, Aras M, Sloey C, Sutherland W, Arai H (2006). Modulation of protein aggregation by polyethylene glycol conjugation: GCSF as a case study. Protein Sci.

[156] Yun Y, Cho YW, Park K (2013). Nanoparticles for oral delivery: targeted nanoparticles with peptidic ligands for oral protein delivery. Adv Drug Deliv Rev.

[157] Cao SJ, Xu S, Wang HM, Ling Y, Dong J, Xia RD (2019). Nanoparticles: oral delivery for protein and peptide drugs. AAPS PharmSciTech.

[158] Allen TM, Cullis PR (2004). Drug delivery systems: entering the mainstream. Science.

[159] Aboubakar M, Couvreur P, Pinto-Alphandary H, Gouritin B, Lacour B, Farinotti R (2000). Insulin-loaded nanocapsules for oral administration: in vitro and in vivo investigation. Drug Dev Res.

[160] Lopes MA, Abrahim BA, Cabral LM, Rodrigues CR, Seiça RM, de Baptista Veiga FJ (2014). Intestinal absorption of insulin nanoparticles: contribution of M cells. Nanomedicine.

[161] Pan Y, Li YJ, Zhao HY, Zheng JM, Xu H, Wei G (2002). Bioadhesive polysaccharide in protein delivery system: chitosan nanoparticles improve the intestinal absorption of insulin in vivo. Int J Pharm.

[162] Chen Z, Han S, Yang X, Xu L, Qi H, Hao G (2020). Overcoming multiple absorption barrier for insulin oral delivery using multifunctional nanoparticles based on chitosan derivatives and hyaluronic acid. Int J Nanomedicine.

[163] Couvreur P, Puisieux F (1993). Nano- and microparticles for the delivery of polypeptides and proteins. Adv Drug Deliv Rev.

[164] Shirangi M, Hennink WE, Somsen GW, van Nostrum CF (2015). Identification and assessment of octreotide acylation in polyester microspheres by LC-MS/MS. Pharm Res.

[165] Werle M, Takeuchi H (2009). Chitosan-aprotinin coated liposomes for oral peptide delivery: development, characterisation and in vivo evaluation. Int J Pharm.

[166] Degim Z, Unal N, Eşsiz D, Abbasoglu U (2004). The effect of various liposome formulations on insulin penetration across Caco-2 cell monolayer. Life Sci.

[167] Rogers JA, Anderson KE (1998). The potential of liposomes in oral drug delivery. Crit Rev Ther Drug Carrier Syst.

[168] Patel A, Cholkar K, Mitra AK (2014). Recent developments in protein and peptide parenteral delivery approaches. Ther Deliv.

[169] Xu W, Ling P, Zhang T (2013). Polymeric micelles, a promising drug delivery system to enhance bioavailability of poorly water-soluble drugs. J Drug Deliv.

[170] Li H, Yan K, Shang Y, Shrestha L, Liao R, Liu F (2015). Folate-bovine serum albumin functionalized polymeric micelles loaded with superparamagnetic iron oxide nanoparticles for tumor targeting and magnetic resonance imaging. Acta Biomater.

[171] Mathot F, van Beijsterveldt L, Préat V, Brewster M, Ariën A (2006). Intestinal uptake and biodistribution of novel polymeric micelles after oral administration. J Control Release.

[172] Kim S, Shi Y, Kim JY, Park K, Cheng JX (2010). Overcoming the barriers in micellar drug delivery: loading efficiency, in vivo stability, and micelle-cell interaction. Expert Opin Drug Deliv.

[173] Mandal TK, Bostanian LA, Graves RA, Chapman SR (2002). Poly(D,L-lactide-co-glycolide) encapsulated poly(vinyl alcohol) hydrogel as a drug delivery system. Pharm Res.

[174] Yeh MK (2000). The stability of insulin in biodegradable microparticles based on blends of lactide polymers and polyethylene glycol. J Microencapsul.

[175] Milstein SJ, Barantsevitch EN, Grechanovski VA, Sarubbi DJ (1996). pH-dependent microspheres from modified soybean protein hydrolysate. J Microencapsul.

[176] Osswald CR, Kang-Mieler JJ (2016). Controlled and extended in vitro release of bioactive anti-vascular endothelial growth factors from a microsphere-hydrogel drug delivery system. Curr Eye Res.

[177] Lee HJ (2002). Protein drug oral delivery: the recent progress. Arch Pharm Res.

[178] Philip AK, Philip B (2010). Colon targeted drug delivery systems: a review on primary and novel approaches. Oman Med J.

[179] Yang L, Chu JS, Fix JA (2002). Colon-specific drug delivery: new approaches and in vitro/in vivo evaluation. Int J Pharm.

[180] Castanho M, Santos N. Peptide Drug Discovery and Development: Translational Research in Academia and Industry. Hoboken, NJ: Wiley; 2011.

[181] Owens DR, Zinman B, Bolli G (2003). Alternative routes of insulin delivery. Diabet Med.

[182] Rubio-Aliaga I, Daniel H (2002). Mammalian peptide transporters as targets for drug delivery. Trends Pharmacol Sci.

[183] Friedrichsen GM, Nielsen CU, Steffansen B, Begtrup M (2001). Model prodrugs designed for the intestinal peptide transporter A synthetic approach for coupling of hydroxy-containing compounds to dipeptides. Eur J Pharm Sci.

[184] Kazi M, Al-Swairi M, Ahmad A, Raish M, Alanazi FK, Badran MM (2019). Evaluation of self-nanoemulsifying drug delivery systems (SNEDDS) for poorly water-soluble talinolol: preparation, in vitro and in vivo assessment. Front Pharmacol.

[185] Jena D, Akila Devi D, Babikir MA (2021). A Review on game-changing approach for the oral administration of lipophilic drug: SEDDS. Res J Pharm Technol.

[186] Mahmood A, Prüfert F, Efiana NA, Ashraf MI, Hermann M, Hussain S (2016). Cell-penetrating self-nanoemulsifying drug delivery systems (SNEDDS) for oral gene delivery. Expert Opin Drug Deliv.

[187] Li P, Nielsen HM, Müllertz A (2016). Impact of lipid-based drug delivery systems on the transport and uptake of insulin across Caco-2 cell monolayers. J Pharm Sci.

[188] Bravo-Alfaro DA, Muñoz-Correa MOF, Santos-Luna D, Toro-Vazquez JF, Cano-Sarmiento C, García-Varela R (2020). Encapsulation of an insulin-modified phosphatidylcholine complex in a self-nanoemulsifying drug delivery system (SNEDDS) for oral insulin delivery. J Drug Deliv Sci Technol.

[189] Karamanidou T, Karidi K, Bourganis V, Kontonikola K, Kammona O, Kiparissides C (2015). Effective incorporation of insulin in mucus permeating self-nanoemulsifying drug delivery systems. Eur J Pharm Biopharm.

[190] Homayun B, Lin X, Choi HJ (2019). Challenges and recent progress in oral drug delivery systems for biopharmaceuticals. Pharmaceutics.

[191] Mlynek GM, Calvo LJ, Robinson JR (2000). Carrier-enhanced human growth hormone absorption across isolated rabbit intestinal tissue. Int J Pharm.

[192] Stern W, Mehta N, Carl S (2013). Oral delivery of peptides by PeptelligenceTM technology. Drug Dev Deliv.

[193] Peptelligence® -- Oral Peptide & Small Molecule Drug Delivery Platform [Internet]. Boonton, NJ: Enteris BioPharma. Available from: https://enterisbiopharma.com/peptelligence/. Accessed August 7, 2022.

[194] Zizzari AT, Pliatsika D, Gall FM, Fischer T, Riedl R (2021). New perspectives in oral peptide delivery. Drug Discov Today.

[195] Lin PY, Chuang EY, Chiu YH, Chen HL, Lin KJ, Juang JH (2017). Safety and efficacy of self-assembling bubble carriers stabilized with sodium dodecyl sulfate for oral delivery of therapeutic proteins. J Control Release.

[196] Tyagi P, Pechenov S, Anand Subramony J (2018). Oral peptide delivery: translational challenges due to physiological effects. J Control Release.

[197] Gleeson JP, Fein KC, Chaudhary N, Doerfler R, Newby AN, Whitehead KA (2021). The enhanced intestinal permeability of infant mice enables oral protein and macromolecular absorption without delivery technology. Int J Pharm.

[198] Yáñez JA, Wang SWJ, Knemeyer IW, Wirth MA, Alton KB (2011). Intestinal lymphatic transport for drug delivery. Adv Drug Deliv Rev.

[199] Abdallah M, Müllertz OO, Styles IK, Mörsdorf A, Quinn JF, Whittaker MR (2020). Lymphatic targeting by albumin-hitchhiking: applications and optimisation. J Control Release.

[200] Deak ST, Csáky TZ (1984). Factors regulating the exchange of nutrients and drugs between lymph and blood in the small intestine. Microcirc Endothelium Lymphatics.

[201] Ikeda I, Imasato Y, Sasaki E, Sugano M (1996). Lymphatic transport of alpha-, gamma- and delta-tocotrienols and alpha-tocopherol in rats. Int J Vitam Nutr Res.

[202] Muranishi S (1997). [Delivery system design for improvement of intestinal absorption of peptide drugs]. Yakugaku Zasshi.

